# Simulation of functional additive and non-additive genetic effects using statistical estimates from quantitative genetic models

**DOI:** 10.1038/s41437-024-00690-5

**Published:** 2024-05-31

**Authors:** Thinh Tuan Chu, Peter Skov Kristensen, Just Jensen

**Affiliations:** 1 Center for Quantitative Genetics and Genomics, Aarhus University, Aarhus, Denmark; 2 Vietnam National University of Agriculture, Faculty of Animal Science, Trâu Quỳ, Gia Lâm, Hanoi, Vietnam

**Keywords:** Animal breeding, Genetic models, Plant breeding

## Abstract

Stochastic simulation software is commonly used to aid breeders designing cost-effective breeding programs and to validate statistical models used in genetic evaluation. An essential feature of the software is the ability to simulate populations with desired genetic and non-genetic parameters. However, this feature often fails when non-additive effects due to dominance or epistasis are modeled, as the desired properties of simulated populations are estimated from classical quantitative genetic statistical models formulated at the population level. The software simulates underlying functional effects for genotypic values at the individual level, which are not necessarily the same as effects from statistical models in which dominance and epistasis are included. This paper provides the theoretical basis and mathematical formulas for the transformation between functional and statistical effects in such simulations. The transformation is demonstrated with two statistical models analyzing individual phenotypes in a single population (common in animal breeding) and plot phenotypes of three-way hybrids involving two inbred populations (observed in some crop breeding programs). We also describe different methods for the simulation of functional effects for additive genetics, dominance, and epistasis to achieve the desired levels of variance components in classical statistical models used in quantitative genetics.

## Introduction

Stochastic simulation software (Pedersen et al. 2009; Faux et al. 2016; Pérez-Enciso et al. 2020; Pook et al. 2020; Gaynor et al. 2021; Chen et al. 2022) has been developed to assist breeders designing cost-effective breeding programs, understanding the impacts of different implementations on genetic gain and inbreeding rates, and validating statistical models used in genetic evaluation. These software packages simulate phenotypes of individuals in populations. An essential feature of this software is the ability to simulate user-desired population properties for the examination of genetic and non-genetic effects. However, this feature fails in commonly used programs for the simulation of non-additive effects due to dominance or epistasis and the inclusion of multiple populations in a breeding program.

With finite locus models, in which the genetic effects of individuals are simulated by finite numbers of quantitative trait loci (QTL), simulation software (Pedersen et al. 2009; Pérez-Enciso et al. 2020; Pook et al. 2020; Gaynor et al. 2021; Chen et al. 2022) samples underlying functional effects of genes (i.e., QTL) from user-defined distributions. These distributions usually derive from statistical estimates from models of real data expressed as (co)variance components or estimated QTL effects. In the absence of non-additive genetic effects and multi-population simulation, simulated populations usually have the properties desired by users because the functional and statistical effects are identical. With dominance and epistasis, however, genetic effects differ between functional and statistical models (Vitezica et al. 2017). Statistical models partly transform the functional effects of dominance and epistasis into additive allele substitution effects of QTL (Vitezica et al. 2013; Duenk et al. 2020). The functional effects of QTL assumed to be unchanged genotypic values are independent of allele frequencies in the population, whereas the statistical effects are substitution effects that also depend on allele frequencies (Falconer and Mackay 1996; Álvarez-Castro and Carlborg 2007; Vitezica et al. 2017). To meet the desired statistical variance components for simulated populations, users must provide the simulation software (Faux et al. 2016; Pook et al. 2020; Chen et al. 2022) with functional genetic effects, which cannot be obtained directly by model estimation with real data.

To our knowledge, no current simulation software or publication shows how functional effects can be sampled when estimates of variance components from classical quantitative genetic statistical models are provided. In an attempt to meet the statistical variance of populations, Karaman et al. (2021) simulated statistical effects of QTL directly instead of functional effects. However, this approach does not guarantee the same functional effects of QTL across populations, as the authors claimed that it would (Karaman et al. 2021). The assumption that the functional effects of QTL are the same, regardless of origin, appears in the literature (Vitezica et al. 2016; Karaman et al. 2021; Poulsen et al. 2022). This assumption could be true if there were no genotype–environment or genotype–genotype interaction, mutation, or multi-allelic QTL states. In the approach proposed by Karaman et al. (2021), how statistical effects are inherited by descendants is unclear. The authors simulated statistical effects for populations in which parents and descendants were already known, as these effects depend on allele frequencies. Thus, the simulation approach (Karaman et al. 2021) may be useful for model evaluation, but not for the simulation of breeding programs, in which selection decisions in the parental generation have consequences for offspring. In other words, the functional effects of QTL are required for the simulation of a breeding scheme, so that the phenotypes of any known genotypes can be simulated independently of allele frequencies.

Many current simulation programs are designed to simulate the data type of individual phenotypes and genotypes (iPGs). The model for the prediction of this data type is based on individuals’ phenotypes and genomic data. In some crop breeding programs, however, hybrids are not genotyped and individual phenotypes for hybrids are not commonly recorded. The data type for these crops consists of the phenotypes of plots that are hybrids of crossings between known-genotype parents from different populations (González-Diéguez et al. 2021; de Jong et al. 2023; Kristensen et al. 2023). The model for the prediction of this data type shows a large difference in the additive genetic variance contributed by the parental populations (Kristensen et al. 2023). The question is thus how the functional effects of QTL can be simulated to achieve desired differences in statistical variance.

Álvarez-Castro and Carlborg (2007) developed a natural and orthogonal interactions (NOIA) model that mathematically translates the relationships between functional and statistical effects and has been used in several studies (Vitezica et al. 2017; Duenk et al. 2020; Wientjes et al. 2023). However, this model has not been used to simulate data to meet specific requirements related to statistical variance components.

This paper describes a method for the simulation of functional QTL effects for additive genetics, dominance, and epistasis to achieve desired variance components in classical statistical models. The simulation method will be demonstrated for two data types: individual phenotypes in a single population, and plot phenotypes involving two inbred populations in a three-way hybrid crop breeding program.

## Assumptions and theory

### Individual phenotype in a single population

The true underlying model for the phenotype of diploid individual $i$ is assumed to be the following:

M.1 $${y}_{i}=\mu +\mathop{\sum }\limits_{j=1}^{{n}_{{qtl}}}{t}_{j,i}^{a}{a}_{j}+\mathop{\sum }\limits_{j=1}^{{n}_{{qtl}}}{t}_{j,i}^{d}{d}_{j}+\mathop{\sum }\limits_{{j}_{{kl}}=1}^{{n}_{{ep}}}{t}_{{kl},i}^{{ep}}{({aa})}_{{j}_{{kl}}}+{e}_{i}$$

where ${y}_{i}$ is the phenotypic value of the *i*^*th*^ individual, $\mu$ is the population mean, and ${e}_{i}$ is the residual environmental effect. Other notations are defined in Table 1. The covariates ${t}_{j,i}^{a}$ and ${t}_{j,i}^{d}$ for QTL genotypes at locus $j$ are:

1 $${t}_{j,i}^{a}=\left\{\begin{array}{c}-1\\ 0\\ 1\end{array}\right.{\rm{;and}}{t}_{j,i}^{d}=\left\{\begin{array}{c}0\\ 1\\ 0\end{array}\right.{\rm{for\; genotype}}\left\{\begin{array}{c}{BB}\\ {Bb}\\ {bb}\end{array}\right.$$

**Table 1:** List of symbols.

Symbol	Definition
	General symbols
${n}_{{qtl}}$	Number of QTL
${a}_{k}$, ${a}_{l}$, ${a}_{j}$	Additive functional effects at QTL *k*, *l* and *j*
${\alpha }_{k}$, ${\alpha }_{l}$, ${\alpha }_{j}$	Additive statistical effects at QTL *k*, *l* and *j* corresponding to ${a}_{k}$, ${a}_{l}$ and ${a}_{j}$
${d}_{j}$, ${d}_{k}$, ${d}_{l}$	Functional dominance value of QTL *k*, *l* and *j*
${\delta }_{j}$, ${\delta }_{k}$, ${\delta }_{l}$	Statistical dominance value of QTL *k*, *l* and *j*. In this paper, ${d}_{x}={\delta }_{x}$
${t}_{j,i}^{a}$, ${t}_{j,i}^{d}$	Covariate of functional additive genetic effects and dominance at QTL *j* for individual *i*
${n}_{{ep}}$	Number of two-way interaction pairs for additive genetic epistasis
${({aa})}_{{j}_{{kl}}}$, ${(\alpha \alpha )}_{{j}_{{kl}}}$	Two-way functional and statistical additive genetic epistatic effect of interaction pair ${j}_{{kl}}$ between loci *k* and *l*. In this paper, ${({aa})}_{{j}_{{kl}}}={(\alpha \alpha )}_{{j}_{{kl}}}$
${t}_{{j}_{{kl}},i}^{{ep}}$, ${h}_{{j}_{{kl}},i}^{{ep}}$	Covariates of functional and statistical epistasis for interaction pair ${j}_{{kl}}$ for individual *i*
${p}_{j}$, ${q}_{j}$	Frequencies of allele B (${p}_{j}$) and allele b (${q}_{j}$) at locus *j*
${p}_{j,{BB}}$, ${p}_{j,{Bb}}$, ${p}_{j,{bb}}$	Frequencies of genotype BB, Bb, and bb at locus *j*
${h}_{j,i}^{a}$, ${h}_{j,i}^{d}$	Covariates of statistical additive genetic effects and dominance at QTL *j* for individual *i*
${{\bf{E}}}_{{\rm{f}},{\rm{kl}}}$, ${{\bf{E}}}_{{\rm{s}},{\rm{kl}}}$	Vectors of functional and statistical effects for QTL *k* and *l*
${{\bf{W}}}_{{\bf{f}}{\boldsymbol{,}}{\bf{kl}}}$, ${{\bf{W}}}_{{\bf{s}}{\boldsymbol{,}}{\bf{kl}}}$	Matrices of covariates linking functional (${{\bf{E}}}_{{\rm{f}},{\rm{kl}}}$) and statistical effects (${{\bf{E}}}_{{\rm{s}},{\rm{kl}}}$)
${\bf{J}}$	A vector of 1 used for positioning elements in constructing matrices ${{\bf{W}}}_{{\rm{f}},{\rm{kl}}}$ and ${{\bf{W}}}_{{\rm{s}},{\rm{kl}}}$
${\bf{u}}$, ${\bf{v}}$, ${\boldsymbol{(}}{\bf{uu}}{\boldsymbol{)}}$	Vectors of statistical additive values, dominance values and epistasis for individuals
${u}_{i}$, ${v}_{i}$, ${({uu})}_{i}$	Statistical additive values, dominance values and epistasis for individual $i$
Variance by individual	Calculation of variance based on the effects of individuals within population. Eg. variance of ${\bf{u}}$, ${\bf{v}}$, ${\boldsymbol{(}}{\bf{uu}}{\boldsymbol{)}}$.
${\sigma }_{A}^{2}$, ${\sigma }_{D}^{2}$, ${\sigma }_{{AA}}^{2}$	Statistical variances of additive genetics, dominance and epistasis
${\sigma }_{{A}_{j}}^{2}$	Statistical additive genetic variance at locus $j$.
Variance by locus	Variance is the sum of variance at all loci. Eg. ${\sigma }_{A}^{2}=\mathop{\sum }\nolimits_{j}^{{n}_{{qtl}}}{\sigma }_{{A}_{j}}^{2}$
${\bf{a}}$, ${\bf{d}}$, $({\bf{aa}}{\boldsymbol{)}}$	Vectors of functional effects for additive genetics, dominance and epistasis affecting phenotypes the same across populations
${{\bf{a}}}^{* }$, ${{\bf{d}}}^{* }$, ${{\boldsymbol{(}}{\bf{aa}}{\boldsymbol{)}}}^{* }$	Prior/starting values for vectors of functional effects ${\bf{a}}$, ${\bf{d}}$ and $({\bf{aa}}{\boldsymbol{)}}$
	Symbols for data type of individual phenotype within a single population
${\sigma }_{{A}^{* }}^{2}$, ${\sigma }_{{D}^{* }}^{2}$, ${\sigma }_{{{AA}}^{* }}^{2}$	Prior/starting values for statistical variances ${\sigma }_{A}^{2}$, ${\sigma }_{D}^{2},$ ${\sigma }_{{AA}}^{2}$
${\boldsymbol{\alpha }}$, ${\boldsymbol{\delta }}$, ${\boldsymbol{(}}{\boldsymbol{\alpha }}{\boldsymbol{\alpha }}{\boldsymbol{)}}$	Vectors of statistical effects for additive genetics, dominance and epistasis. In this paper, ${\boldsymbol{\delta }}={\bf{d}}$, and $({\boldsymbol{\alpha }}{\boldsymbol{\alpha }}{\boldsymbol{)}}=({\bf{aa}}{\boldsymbol{)}}$
${{\boldsymbol{\alpha }}}^{{\boldsymbol{* }}}$, ${{\boldsymbol{\delta }}}^{{\boldsymbol{* }}}$, ${({\boldsymbol{\alpha }}{\boldsymbol{\alpha }}{\boldsymbol{)}}}^{* }$	Prior/starting values for vectors of statistical effects ${\boldsymbol{\alpha }}$, ${\boldsymbol{\delta }}$ and ${\boldsymbol{(}}{\boldsymbol{\alpha }}{\boldsymbol{\alpha }}{\boldsymbol{)}}$
	Symbols for plot phenotype of three-way hybrids by crossing two populations
${\sigma }_{A,1}^{2}$, ${\sigma }_{A,2}^{2}$	Statistical additive genetic variances of three-way hybrid plot that are explained by the genotype of the inbred population 1, and 2
${\sigma }_{D,3}^{2}$, ${\sigma }_{{AA},3}^{2}$	Statistical dominance and epistatic variances of three-way hybrid plot explained by the genotypes of inbred populations
${\sigma }_{{A}^{* },1}^{2}$, ${\sigma }_{{A}^{* },2}^{2}$, ${\sigma }_{{D}^{* },3}^{2}$, ${\sigma }_{{{AA}}^{* },3}^{2}$	Prior/starting values for statistical variances ${\sigma }_{A,1}^{2}$, ${\sigma }_{A,2}^{2}$, ${\sigma }_{D,3}^{2}$, and ${\sigma }_{{AA},3}^{2}$
${{\boldsymbol{\alpha }}}_{1}$, ${{\boldsymbol{\alpha }}}_{2}$	Vectors of statistical additive genetic effects on three-way hybrid phenotype determined by the genotypes of parents from populations 1 and 2
${{\boldsymbol{\delta }}}_{3}$, ${({\boldsymbol{\alpha }}{\boldsymbol{\alpha }}{\boldsymbol{)}}}_{3}$	Vectors of statistical dominance and epistasis. In this paper, ${{\boldsymbol{\delta }}}_{3}={\bf{d}}$, and ${({\boldsymbol{\alpha }}{\boldsymbol{\alpha }}{\boldsymbol{)}}}_{3}=({\bf{aa}}{\boldsymbol{)}}$
${{\boldsymbol{\alpha }}}_{1}^{* }$, ${{\boldsymbol{\alpha }}}_{2}^{* }$, ${{\boldsymbol{\delta }}}_{3}^{* }$, ${({\boldsymbol{\alpha }}{\boldsymbol{\alpha }}{\boldsymbol{)}}}_{3}^{* }$	Prior/starting values for vectors of statistical effects ${{\boldsymbol{\alpha }}}_{1}$, ${{\boldsymbol{\alpha }}}_{2}$, ${{\boldsymbol{\delta }}}_{3}$ and ${({\boldsymbol{\alpha }}{\boldsymbol{\alpha }}{\boldsymbol{)}}}_{3}$

The covariate $${t}_{{j}_{{kl}},i}^{{ep}}={t}_{l,i}^{a}* {t}_{k,i}^{a}$$.

Model M.1 is assumed to be the underlying functional or genotypic model for simulating phenotypes of individual genotypes just as done in popular simulation software like AlphaSim (Gaynor et al. 2021) and other studies (Pook et al. 2020; Duenk et al. 2021). In this paper, a simplified model of epistasis that restricts epistatic interactions to pairs of loci for additive × additive is assumed. Higher-order interactions and the epistasis of additive × dominance and dominance × dominance interactions are often difficult to estimate with real data (González-Diéguez et al. 2021), so that the inputs for these interactions may be not available for simulations. However, the theory to be presented is general and can be extended to such more complicated models.

Model M.1 is not recommended for estimation of parameters, or selection because effects could not be estimated independently. For example, the mean, additive and dominance effects could be confounded. This model is similar to genotypic or biological models in literature (Su et al. 2012; Vitezica et al. 2013; Jiang and Reif 2015). The classical quantitative genetic model corresponding to M.1 is:

M.2 $${y}_{i}=\mu +\mathop{\sum }\limits_{j=1}^{{n}_{{qtl}}}{h}_{j,i}^{a}{\alpha }_{j}+\mathop{\sum }\limits_{j=1}^{{n}_{{qtl}}}{h}_{j,i}^{d}{\delta }_{j}+\mathop{\sum }\limits_{{j}_{{kl}}=1}^{{n}_{{ep}}}{h}_{{j}_{{kl}},i}^{{ep}}{(\alpha \alpha )}_{{j}_{{kl}}}+{e}_{i}$$

where the covariates ${h}_{j,i}^{a}$ and ${h}_{j,i}^{d}$ for the QTL genotype of individual *i* at locus $j$ can be calculated using allele frequencies (${p}_{j}$ and ${q}_{j}$):

2 $${h}_{j,i}^{a}=\left\{\begin{array}{c}(0-2{q}_{j})\\ (1-2{q}_{j})\\ (2-2{q}_{j})\end{array}\right.{\rm{;and}}{h}_{j,i}^{d}=\left\{\begin{array}{c}-2{q}_{j}^{2}\\ 2{p}_{j}{q}_{j}\\ -2{p}_{j}^{2}\end{array}\right.{\rm{for\; genotype}}\left\{\begin{array}{c}{BB}\\ {Bb}\\ {bb}\end{array}\right.$$

or genotype frequencies (${p}_{j,{BB}}$, ${p}_{j,{Bb}}$, and ${p}_{j,{bb}}$):

3 $${h}_{j,i}^{a}=\left\{\begin{array}{c}\left(0-{p}_{j,{Bb}}-2{p}_{j,{bb}}\right)\\ \left(1-{p}_{j,{Bb}}-2{p}_{j,{bb}}\right)\\ \left(2-{p}_{j,{Bb}}-2{p}_{j,{bb}}\right)\end{array}\right.{\rm{and}}{h}_{j,i}^{d}=\left\{\begin{array}{c}\frac{-2{p}_{j,{Bb}}{p}_{j,{bb}}}{{p}_{j,{BB}}+{p}_{j,{bb}}-{({p}_{j,{BB}}-{p}_{j,{bb}})}^{2}}\\ \frac{4{p}_{j,{BB}}{p}_{j,{bb}}}{{p}_{j,{BB}}+{p}_{j,{bb}}-{({p}_{j,{BB}}-{p}_{j,{bb}})}^{2}}\\ \frac{-2{p}_{j,{BB}}{p}_{j,{Bb}}}{{p}_{j,{BB}}+{p}_{j,{bb}}-{({p}_{j,{BB}}-{p}_{j,{bb}})}^{2}}\end{array}\right.{\rm{for\; genotype}}\left\{\begin{array}{c}{BB}\\ {Bb}\\ {bb}\end{array}\right.$$

Best linear unbiased prediction (BLUP) is often used to estimate the parameters of model M.2 at the single nucleotide polymorphism (SNP) level, thus the model is known as snpBLUP model. To be consistent with previous studies (Álvarez-Castro and Carlborg 2007; Vitezica et al. 2013; Vitezica et al. 2017), M.2 is called a statistical model. Model M.2 has orthogonal properties. Álvarez-Castro and Carlborg (2007) and Vitezica et al. (2017) describe the importance of these properties, which enable the estimation of each genetic effect independently. In this paper, the term “statistical effects” [*α*, *δ* and (*αα*)] denotes effects from statistical models, and “functional effects” [*a*, *d* and (*aa*)] refers to the underlying functional effects of genes at the individual level.

The covariate ${h}_{j,i}^{a}$ in Eqs. 2 and 3 is equivalent, regardless of the Hardy–Weinberg equilibrium (HWE) status of the population. The covariate ${h}_{j,i}^{d}$ is equivalent in Eqs. 2 and 3 when the population is in HWE, but must be calculated using Eq. 3 when the population is not in HWE. The covariate ${h}_{{j}_{{kl}},i}^{{ep}}$ for the genotype of individual *i* for interaction pair ${j}_{{kl}}$ between loci *k* and *l* is calculated as:

4 $${h}_{{j}_{{kl}},i}^{{ep}}={h}_{l,i}^{a}* {h}_{k,i}^{a}$$

Álvarez-Castro and Carlborg (2007) proposed NOIA model to translate between the functional (M.1) and statistical effects (M.2):

5 $${{\bf{W}}}_{{\rm{s}}}{{\bf{E}}}_{{\rm{s}}}={{\bf{W}}}_{{\rm{f}}}{{\bf{E}}}_{{\rm{f}}}$$

where ${{\bf{E}}}_{{\rm{s}}}$ and ${{\bf{E}}}_{{\rm{f}}}$ are the vectors of the statistical and functional genetic effects, respectively, and ${{\bf{W}}}_{{\rm{s}}}$ and ${{\bf{W}}}_{{\rm{f}}}$ are the genetic-effect design matrices linking these effects to genotypes. The elements of **W** are the covariates (coefficients) of the genetic effects present for each genotype.

As an example, we show the conversion of the functional effects in M.1 to statistical effects at loci *k* and *l*:

6 $${{\bf{E}}}_{{\rm{s}},{\rm{kl}}}={{\bf{W}}}_{{\rm{s}},{\rm{kl}}}^{-1{\rm{left}}}{{\bf{W}}}_{{\rm{f}},{\rm{kl}}}{{\bf{E}}}_{{\rm{f}},{\rm{kl}}}$$

Vectors ${{\bf{E}}}_{{\rm{f}},{\rm{kl}}}$ and ${{\bf{E}}}_{{\rm{s}},{\rm{kl}}}$ have a dimension of 6 × 1:

$${{\bf{E}}}_{{\rm{f}},{\rm{kl}}}{\boldsymbol{=}}{\left[\begin{array}{cccccc}1 & {a}_{k} & {a}_{l} & {d}_{k} & {d}_{l} & {({aa})}_{{kl}}\end{array}\right]}^{{\boldsymbol{{\prime} }}}$$

$${\rm{and}}\,{{\bf{E}}}_{{\rm{s}},{\rm{kl}}}{\boldsymbol{=}}{\left[\begin{array}{cccccc}\mu & {\alpha }_{k} & {\alpha }_{l} & {\delta }_{k} & {\delta }_{l} & {(\alpha \alpha )}_{{kl}}\end{array}\right]}^{\prime}$$

Matrices ${{\bf{W}}}_{{\rm{f}},{\rm{kl}}}$ and ${{\bf{W}}}_{{\rm{s}},{\rm{kl}}}$ have a dimension of 9 × 6 (rows x columns):

$${{\bf{W}}}_{{\rm{f}},{\rm{kl}}}=\left[\begin{array}{cccccc}{\boldsymbol{1}} & {\bf{J}}\otimes {{\bf{t}}}_{{\rm{k}}}^{{\rm{a}}} & {{\bf{t}}}_{{\rm{l}}}^{{\rm{a}}}\otimes {\bf{J}} & {\bf{J}}\otimes {{\bf{t}}}_{{\rm{k}}}^{{\rm{d}}} & {{\bf{t}}}_{{\rm{l}}}^{{\rm{d}}}\otimes {\bf{J}} & {{\bf{t}}}_{{\rm{l}}}^{{\rm{a}}}\otimes {{\bf{t}}}_{{\rm{k}}}^{{\rm{a}}}\end{array}\right]$$

$${\rm{and}}\,{{\bf{W}}}_{{\rm{s}},{\rm{kl}}}=\left[\begin{array}{cccccc}{\boldsymbol{1}} & {\bf{J}}\otimes {{\bf{h}}}_{{\rm{k}}}^{{\rm{a}}} & {{\bf{h}}}_{{\rm{l}}}^{{\rm{a}}}\otimes {\bf{J}} & {\bf{J}}\otimes {{\bf{h}}}_{{\rm{k}}}^{{\rm{d}}} & {{\bf{h}}}_{{\rm{l}}}^{{\rm{d}}}\otimes {\bf{J}} & {{\bf{h}}}_{{\rm{l}}}^{{\rm{a}}}\otimes {{\bf{h}}}_{{\rm{k}}}^{{\rm{a}}}\end{array}\right]$$

where ${\bf{J}}$ is a 3 × 1 vector with all elements of 1. The elements of the 3 × 1 vector ${{\bf{t}}}_{{\rm{x}}}^{{\rm{a}}}$, ${{\bf{t}}}_{{\rm{x}}}^{{\rm{d}}}$, ${{\bf{h}}}_{{\rm{x}}}^{{\rm{a}}}$, and ${{\bf{h}}}_{{\rm{x}}}^{{\rm{d}}}$ are covariates for ${t}_{x}^{a}$, ${t}_{x}^{d}$ (Eq. 1), ${h}_{x}^{a}$, and ${h}_{x}^{d}$ (Eq. 3), respectively. For example, $${{\bf{t}}}_{{\rm{x}}}^{{\rm{a}}}={\left[\begin{array}{ccc}-1 & 0 & 1\end{array}\right]}^{{\prime} }$$. ${\boldsymbol{\otimes }}$ denotes the Kronecker product. Vector ${\boldsymbol{1}}$ has a dimension of 9 (or number of rows of matrix ${\bf{W}}$).

In Eq. 6, ${{\bf{W}}}_{{\rm{s}},{\rm{kl}}}^{-1{\rm{left}}}$ is the left inverse of ${{\bf{W}}}_{{\rm{s}},{\rm{kl}}}$. We calculate ${{\bf{W}}}_{{\rm{s}},{\rm{kl}}}^{-1{\rm{left}}}{\boldsymbol{=}}{{\boldsymbol{(}}{{\bf{W}}}_{{\rm{s}},{\rm{kl}}}^{{\boldsymbol{{\prime} }}}{{\bf{W}}}_{{\rm{s}},{\rm{kl}}}{\boldsymbol{)}}}^{-1}{{\bf{W}}}_{{\rm{s}},{\rm{kl}}}^{{\boldsymbol{{\prime} }}}$. Thus, Eq. 6 becomes:

7 $${{\bf{E}}}_{{\rm{s}},{\rm{kl}}}={({{\bf{W}}}_{{\rm{s}},{\rm{kl}}}^{{\prime} }{{\bf{W}}}_{{\rm{s}},{\rm{kl}}})}^{-1}{{\bf{W}}}_{{\rm{s}},{\rm{kl}}}^{{\prime} }{{\bf{W}}}_{{\rm{f}},{\rm{kl}}}{{\bf{E}}}_{{\rm{f}},{\rm{kl}}}$$

Note that the formulas from Vitezica et al. (2017) and Duenk et al. (2020) differ slightly from ours. They included typographical errors in the order of Kronecker product matrices in the calculation of ${{\bf{W}}}_{{\rm{s}},{\rm{kl}}}$ (Vitezica et al. 2017; Duenk et al. 2020). They also included the diagonal matrix of frequencies (Vitezica et al. 2017; Duenk et al. 2020), which is actually already canceled out of the equation. Appendix 1 has an R script example to test the NOIA formula numerically with and without the diagonal frequency matrix.

Without the interaction of epistasis with dominance, the statistical effect of epistasis is equal to the functional effect: ${\left(\alpha \alpha \right)}_{{kl}}={\left({aa}\right)}_{{kl}}$ assuming QTLs are in linkage equilibrium (LE). The statistical effect of the dominance of QTL *k* is also equal to the functional effect: ${\delta }_{k}={d}_{k}$. The dominance and epistatic effects of a QTL are unchanged, but the variance of these effects differs because the covariates of the functional (${t}_{k}$ in M.1) and statistical (${h}_{k}$ in M.2) effects differ. In contrast, the functional and statistical effects differ for additive genetics:

$${\alpha }_{k}={a}_{k}+\left(1-2{q}_{k}\right){d}_{k}+{(\alpha \alpha )}_{{kl}}^{k}$$

where ${(\alpha \alpha )}_{{kl}}^{k}$ is the term that epistasis contributes to the additive effect of QTL $k$. Formula Eq.7 gives only ${\alpha }_{k}$, but the term ${(\alpha \alpha )}_{{kl}}^{k}$ can be calculated separately using a similar formula with $${{\bf{E}}}_{{\rm{f}},{\rm{kl}}}{\boldsymbol{=}}{\left[\begin{array}{cccccc}1 & 0 & 0 & 0 & 0 & {({aa})}_{{kl}}\end{array}\right]}^{{\boldsymbol{{\prime} }}}$$. The vector ${{\bf{E}}}_{{\rm{s}},{\rm{kl}}}$ becomes $${{\bf{E}}}_{{\rm{s}},{\rm{kl}}}{\boldsymbol{=}}{\left[\begin{array}{cccccc}\mu & {(\alpha \alpha )}_{{kl}}^{k} & {(\alpha \alpha )}_{{kl}}^{l} & 0 & 0 & {(\alpha \alpha )}_{{kl}}\end{array}\right]}^{{\boldsymbol{{\prime} }}}$$. Duenk et al. (2020) take a similar approach to the calculation of ${(\alpha \alpha )}_{{kl}}^{k}$. In this paper, a QTL is assumed to interact with only one other QTL. If a QTL interacted with more than one QTL, the formula would be ${\alpha }_{k}={a}_{k}+\left(1-2{q}_{k}\right){d}_{k}+\mathop{\sum }\nolimits_{z=1}^{{n}_{z}}{\left(\alpha \alpha \right)}_{z}^{k}$, where ${n}_{z}$ is the number of loci with which QTL $k$ interacts (Duenk et al. 2020).

In this paper, genetic variance is calculated in two approximated ways: by individual and by locus. Each individual $i$ in a population has functional values of additive genetics ${u}_{i}^{* }=\mathop{\sum }\nolimits_{j=1}^{{n}_{{qtl}}}{t}_{j,i}^{a}{a}_{j}$, dominance ${v}_{i}^{* }=\mathop{\sum }\nolimits_{j=1}^{{n}_{{qtl}}}{t}_{j,i}^{d}{d}_{j}$ and epistasis ${({uu})}_{i}^{* }=\mathop{\sum }\nolimits_{{j}_{{kl}}=1}^{{n}_{{ep}}}{t}_{{j}_{{kl}},i}^{{ep}}{\left({aa}\right)}_{{j}_{{kl}}}$ as in M.1, or statistical values of ${u}_{i}=\mathop{\sum }\nolimits_{j=1}^{{n}_{{qtl}}}{h}_{j,i}^{a}{\alpha }_{j}$, ${v}_{i}=\mathop{\sum }\nolimits_{j=1}^{{n}_{{qtl}}}{h}_{j,i}^{d}{\delta }_{j}$ and ${({uu})}_{i}=\mathop{\sum }\nolimits_{{j}_{{kl}}=1}^{{n}_{{ep}}}{h}_{{j}_{{kl}},i}^{{ep}}{(\alpha \alpha )}_{{j}_{{kl}}}$ as in M.2. The calculation of statistical additive variance ${\sigma }_{A}^{2}$ by individual, for example, would be the variance of ${u}_{i}$ values. Alternatively, the calculation of the variance by locus is the sum of variance from all loci. For each locus $j$, the allele frequency and QTL effect were known, enabling the calculation of variance. For example, the statistical additive variance ${\sigma }_{{A}_{j}}^{2}$ at locus $j$ in HWE is

8 $$\begin{array}{l}{\sigma }_{{A}_{j}}^{2}={[{\alpha }_{j}(0-2{q}_{j})-{\mu }_{{\alpha }_{j}}]}^{2}{p}_{j}^{2}+{[{\alpha }_{j}(1-2{q}_{j})-{\mu }_{{\alpha }_{j}}]}^{2}2{p}_{j}{q}_{j}\\\qquad\quad+\,{[{\alpha }_{j}(2-2{q}_{j})-{\mu }_{{\alpha }_{j}}]}^{2}{q}_{j}^{2}\end{array}$$

where $${\mu }_{{\alpha }_{j}}={\alpha }_{j}(0-2{q}_{j}){p}_{j}^{2}+{\alpha }_{j}(1-2{q}_{j})2{p}_{j}{q}_{j}+{\alpha }_{j}(2-2{q}_{j}){q}_{j}^{2}$$

or $${\mu }_{{\alpha }_{j}}={\alpha }_{j}({q}_{j}-{p}_{j})$$

Simplification of this equation leads to ${\sigma }_{{A}_{j}}^{2}=2{p}_{j}{q}_{j}{\alpha }_{j}^{2}$, which is equivalent to the formula of Vitezica et al. (2013). The statistical additive variance ${\sigma }_{A}^{2}$ by locus is then equal to the sum of ${\sigma }_{{A}_{j}}^{2}$ from all loci. The calculation of variance by locus assumes that there is no covariance between loci, i.e., that all loci are in LE. Using similar derivations, we can calculate variance for dominance and epistasis.

In practice, snpBLUP model (i.e., M.2) is computationally tedious when QTL genotypes are unknown, epistatic interaction pairs are unknown, or ${n}_{{ep}}$ is huge (Vitezica et al. 2017). For this reason, genomic BLUP (GBLUP) models at the individual level are commonly used. In matrix form, GBLUP models predict individual genetic values as (Vitezica et al. 2017):

M.3 $${\bf{y}}={\bf{1}}{\rm{\mu }}+{\bf{Zu}}+{\bf{Zv}}+{\bf{Z}}({\bf{uu}})+{\bf{e}}$$

where ${\bf{y}}$ is the vector of individual phenotypes; ${\rm{\mu }}$ is the population mean; ${\bf{Z}}$ is a design matrix relating individuals to genetic effects; ${\bf{u}}$ is the vector of breeding values ${\bf{u}} \sim {\bf{N}}\left({\bf{0}}{\boldsymbol{,}}\,{{\bf{G}}}_{{\rm{A}}}{\sigma }_{A}^{2}\right)$, where the genomic relationship matrix for additive genetics (${{\bf{G}}}_{{\rm{A}}}$) is constructed as: ${{\bf{G}}}_{{\rm{A}}}{\boldsymbol{=}}\frac{{{\bf{H}}}_{{\rm{a}}}{{\bf{H}}}_{{\rm{a}}}^{{\boldsymbol{{\prime} }}}}{{\rm{tr}}({{\bf{H}}}_{{\rm{a}}}{{\bf{H}}}_{{\rm{a}}}^{{\boldsymbol{{\prime} }}})/n}$ where ${{\bf{H}}}_{{\rm{a}}}$ is a matrix with *n* rows (number of individuals) and *n* columns (number of markers or QTL in this paper), and elements of ${{\bf{H}}}_{{\rm{a}}}$ are as ${h}_{i,j}^{a}$ in Eq. 2 for individual *i* at QTL *j*. Vector ${\bf{v}}$ is the vector of dominance values ${\bf{v}} \sim {\bf{N}}\left({\bf{0}}{\boldsymbol{,}}{{\bf{G}}}_{{\rm{D}}}{\sigma }_{D}^{2}\right)$, where the genomic relationship matrix for dominance (${{\bf{G}}}_{{\rm{D}}}$) is constructed as: ${{\bf{G}}}_{{\rm{D}}}{\boldsymbol{=}}\frac{{{\bf{H}}}_{{\rm{d}}}{{\bf{H}}}_{{\rm{d}}}^{{\boldsymbol{{\prime} }}}}{{\rm{tr}}({{\bf{H}}}_{{\rm{d}}}{{\bf{H}}}_{{\rm{d}}}^{{\boldsymbol{{\prime} }}})/{\rm{n}}}$, where ${{\bf{H}}}_{{\rm{d}}}$ is a matrix with the same dimension as ${{\bf{H}}}_{{\rm{a}}}$, and elements of ${{\bf{H}}}_{{\rm{d}}}$ are as ${h}_{i,j}^{d}$ in Eq. 3 for individual *i* at QTL *j*. Vector $\left({\bf{uu}}\right)$ is the vector of additive × additive epistasis $\left({\bf{uu}}\right) \sim {\bf{N}}\left({\bf{0}}{\boldsymbol{,}}\,{{\bf{G}}}_{{\rm{AA}}}{\sigma }_{{AA}}^{2}\right)$, where the genomic relationship matrix for epistasis (${{\bf{G}}}_{{\rm{AA}}}$) is constructed as: ${{\bf{G}}}_{{\rm{AA}}}=\frac{{{\bf{G}}}_{{\rm{A}}}\odot {{\bf{G}}}_{{\rm{A}}}}{{\rm{tr}}({{\bf{G}}}_{{\rm{A}}}\odot {{\bf{G}}}_{{\rm{A}}})/n}$; ${\odot}$ is the Hadamard product. ${{\bf{G}}}_{{\rm{AA}}}$ is an approximation of the relationship matrix for epistasis (Jiang and Reif 2015; Vitezica et al. 2017). Model M.2 and M.3 are equivalent statistical models when ${n}_{{ep}}$ is very large (Jiang and Reif 2015).

The theory presented so far is based on the assumption that the iPG data type is known or needed. Model M.2, M.3 and the use of frequencies in the calculation of statistical effects involve single populations. This data type and models are often used in simulations of the breeding of purebred animal populations. In crop breeding, however, individual phenotypes are often not observed, but plot phenotypes of hybrids are of interest. In the next section we present the theory underlying the conversion between functional and statistical effects using the NOIA model with data from a three-way hybrid crop breeding program.

### Plot phenotype of three-way hybrids obtained by the crossing of two populations

In three-way hybrid crop breeding, hybrids are individual plants from the same mating of an inbred line with a two-way hybrid (Kristensen et al. 2023), and they may have different genotypes. The inbred lines are from population 1, whereas two-way hybrids are produced by mating two other inbred lines from population 2. This data type has plot phenotypes (of three-way hybrids) and genotypes (of inbred lines; pPGs) in populations 1 and 2. In practice, each hybrid could have several plots as replicates. In this paper, we assume that pPG data have single plots per hybrid, as environmental effects are not of interest. We also assume an infinite number of individual plants within a plot, given the observation in most crop breeding that plots consist of thousands of individual plants. The model of three-way hybrid plot phenotype $i3$ based on the parental genotypes is

M.4 $${y}_{i3}=\mu +\mathop{\sum }\limits_{j=1}^{{n}_{{qtl}}}\frac{1}{2}({t}_{j,i1}+{t}_{j,i2}){a}_{j}+\mathop{\sum }\limits_{j=1}^{{n}_{{qtl}}}{t}_{j,i3}^{d}{d}_{j}+\mathop{\sum }\limits_{{j}_{{kl}}=1}^{{n}_{{epi}}}{t}_{{kl},i3}^{{ep}}{({aa})}_{{j}_{{kl}}}+{e}_{i3}$$

where ${y}_{i3}$ is the plot phenotype of the three-way hybrids, ${e}_{i3}$ is the plot residual term, and ${t}_{j,i1}$ is the genotype of the inbred-line parent of the three-way hybrids from population 1 at locus $j$ (${t}_{j,i1}=-1;0;1$ for BB; Bb; bb genotypes). Similarly, ${t}_{j,i2}$ is the genotype of the two-way hybrid parent of the three-way hybrids (–1; 0; 1). The individual genotype of the two-way hybrid is assumed to be known in M.4. The covariate for dominance at locus $j$ is:

9 $${t}_{j,i3}^{d}=\frac{1}{4}\left[\left(1-{t}_{j,i1}\right)\left({t}_{j,i2}+1\right)+\left({t}_{j,i1}+1\right)\left(1-{t}_{j,i2}\right)\right]$$

The covariate for the epistatic effect of interaction pair ${j}_{{kl}}$ between loci k and l is: ${t}_{{j}_{{kl}},i3}^{{ep}}=\frac{1}{2}({t}_{l,i1}+{t}_{l,i2})* \frac{1}{2}({t}_{k,i1}+{t}_{k,i2})$.

For the genetic evaluation of inbred lines in a three-way hybrid crop breeding program, the statistical GBLUP model is assumed as (González-Diéguez et al. 2021; Kristensen et al. 2023):

M.5 $${{\bf{y}}}_{3}={\bf{1}}{\rm{\mu }}+{{\bf{Z}}}_{{u}_{1}}{{\bf{u}}}_{1}+\left({{\bf{Z}}}_{{{\rm{u}}}_{2,{\rm{a}}}}+{{\bf{Z}}}_{{{\rm{u}}}_{2,{\rm{b}}}}\right){{\bf{u}}}_{2}+{{\bf{Z}}}_{{{\rm{v}}}_{3}}{{\bf{v}}}_{3}+{{\bf{Z}}}_{{({\rm{uu}})}_{3}}{({\bf{uu}})}_{3}+{{\bf{e}}}_{3}$$

where ${{\bf{y}}}_{3}$ is the vector of the three-way hybrid plot phenotype and ${{\bf{u}}}_{1}$ is the vector of breeding values for inbred parental lines in population 1: ${{\bf{u}}}_{1} \sim {\bf{N}}({\bf{0}}{\boldsymbol{,}}\,{{\bf{G}}}_{{{\rm{A}}}_{1}}{\sigma }_{A,1}^{2})$, where the genomic relationship for additive genetics (${{\bf{G}}}_{{{\rm{A}}}_{1}}$) is constructed based on the genotypes of the parental lines in population 1, in the same way as ${{\bf{G}}}_{{\rm{A}}}$ in M.3. Similarly, ${{\bf{u}}}_{2}$ is the vector of breeding values for inbred grandparental lines in population 2: ${{\bf{u}}}_{2} \sim {\bf{N}}({\bf{0}}{\boldsymbol{,}}\,{{\bf{G}}}_{{{\rm{A}}}_{2}}{\sigma }_{A,2}^{2})$. Vector ${{\bf{v}}}_{3}$ is the vector of the three-way hybrid dominance values: ${{\bf{v}}}_{3} \sim {\bf{N}}({\bf{0}}{\boldsymbol{,}}\,{{\bf{G}}}_{{{\rm{D}}}_{3}}{\sigma }_{D,3}^{2})$, where the genomic relationship for dominance (${{\bf{G}}}_{{{\rm{D}}}_{3}}$) is constructed as: ${{\bf{G}}}_{{{\rm{D}}}_{3}}{\boldsymbol{=}}\frac{{{\bf{H}}}_{{{\rm{d}}}_{3}}{{\bf{H}}}_{{{\rm{d}}}_{3}}^{{\boldsymbol{{\prime} }}}}{{\rm{tr}}({{\bf{H}}}_{{{\rm{d}}}_{3}}{{\bf{H}}}_{{{\rm{d}}}_{3}}^{{\boldsymbol{{\prime} }}})/{\rm{n}}}$, where ${{\bf{H}}}_{{{\rm{d}}}_{3}}$ is a matrix with the dimension of *n* rows (number of three-way hybrids, or plots in this paper) and *n* columns (number of QTL). Here, three-way hybrid plot ${i}_{3}$ is the offspring of inbred line ${i}_{1}$ from population 1 and two-way hybrid ${i}_{2}$. The parents of the two-way hybrid are ${i}_{2,a}$ and ${i}_{2,b}$. The element ${h}_{j,i3}^{d}$ of ${{\bf{H}}}_{{{\rm{d}}}_{3}}$ for three-way hybrid ${i}_{3}$ is calculated as:

10 $$\begin{array}{l}{h}_{j,i3}^{d}=\frac{1}{2}\left(1-{t}_{j,i1}\right)\left({t}_{j,i2}+1\right){q}_{j,1}{p}_{j,2}+\frac{1}{2}\left({t}_{j,i1}+1\right)\left(1-{t}_{j,i2}\right){p}_{j,1}{q}_{j,2}\\\qquad\quad-\frac{1}{2}\left(1-{t}_{j,i1}\right)\left(1-{t}_{j,i2}\right){q}_{j,1}{q}_{j,2}-\frac{1}{2}\left({t}_{j,i1}+1\right)\left({t}_{j,i2}+1\right){p}_{j,1}{p}_{j,2}\end{array}$$

where ${t}_{j,i1}$ is the genotype of inbred line ${i}_{1}$ at locus $j$: ${t}_{j,i2}=\frac{1}{2}\left({t}_{j,i2a}+{t}_{j,i2b}\right)$, where ${t}_{j,i2a}$ and ${t}_{j,i2b}$ are genotypes of inbred lines ${i}_{2a}$ and ${i}_{2b}$ at locus $j$ (${t}_{j}=-1;0;1$ for BB; Bb; bb genotypes); ${p}_{j,1}$ and ${p}_{j,2}$ are allele frequencies of *B* in populations 1 and 2, respectively; and ${q}_{j,1}$ and ${q}_{j,2}$ are allele frequencies of *b* in populations 1 and 2, respectively. Elements of dominance relationship matrices in González-Diéguez et al. (2021) and Kristensen et al. (2023) can be calculated using Eq.10. Vector ${{\boldsymbol{(}}{\bf{uu}}{\boldsymbol{)}}}_{3}$ is the vector of additive × additive epistasis of three-way hybrids ${{\boldsymbol{(}}{\bf{uu}}{\boldsymbol{)}}}_{3} \sim {\bf{N}}\left({\bf{0}}{\boldsymbol{,}}\,{{\bf{G}}}_{{{\rm{AA}}}_{3}}{\sigma }_{{AA},3}^{2}\right)$, where the genomic relationship for epistasis (${{\bf{G}}}_{{{\rm{AA}}}_{3}}$) is constructed as: ${{\bf{G}}}_{{{\rm{AA}}}_{3}}=\frac{{{\bf{G}}}_{{{\rm{A}}}_{3}}\odot {{\bf{G}}}_{{{\rm{A}}}_{3}}}{{\boldsymbol{tr}}({{\bf{G}}}_{{{\rm{A}}}_{3}}\odot {{\bf{G}}}_{{{\rm{A}}}_{3}})/n}$; ⊙ is the Hadamard product and ${{\bf{G}}}_{{{\rm{A}}}_{3}}$ is the genomic relationship for additive genetics, constructed similarly as ${{\bf{G}}}_{{\rm{A}}}$ but based on parental-line genotype means, e.g., element ${h}_{j,i3}^{a}$ of ${{\bf{H}}}_{{{\rm{a}}}_{3}}$ for ${{\bf{G}}}_{{{\rm{A}}}_{3}}$ calculation is:

$${h}_{j,i3}^{a}=\frac{1}{2}({t}_{j,i1}-2{q}_{j,1})+\frac{1}{4}\left({t}_{j,i2a}+{t}_{j,i2b}-4{q}_{j,2}\right).$$

Matrices ${{\bf{Z}}}_{x}$ are design matrices. Appendix 2 contains a numerical example illustrating M.5.

Model M.5 is a version of the model of (González-Diéguez et al. 2021; Kristensen et al. 2023), but with no environmental effect or additive × additive epistasis within populations. It assumes different statistical additive effects ${{\boldsymbol{\alpha }}}_{1}$ and ${{\boldsymbol{\alpha }}}_{2}$ for QTL from populations 1 and 2, but we assume the same functional effect ${\bf{a}}$ for the additive genetics across populations in M.4. The functional effects for dominance ${\bf{d}}$ and epistasis ${\boldsymbol{(}}{\bf{aa}}{\boldsymbol{)}}$ are also assumed to be the same across populations in M.4.

To convert from functional to statistical effects, we apply the NOIA formula (Eq. 5). Here, we show the construction of ${{\bf{W}}}_{{\rm{f}},{\rm{kl}}}$ and ${{\bf{W}}}_{{\rm{s}},{\rm{kl}}}$ matrices to convert functional effects ${{\bf{E}}}_{{\rm{f}},{\rm{kl}}}$ in M.4 to statistical effects ${{\bf{E}}}_{{\rm{s}},{\rm{k}}{\bf{l}}}$ at loci *k* and *l* in M.5. Vectors ${{\bf{E}}}_{{\rm{f}},{\rm{kl}}}$ and ${{\bf{E}}}_{{\rm{s}},{\rm{kl}}}$ have the dimension of 8 × 1:

$${{\bf{E}}}_{{\rm{f}},{\rm{kl}}}{\boldsymbol{=}}{\left[\begin{array}{cccccccc}1 & {a}_{k} & {a}_{l} & {a}_{k} & {a}_{l} & {d}_{k} & {d}_{l} & {({aa})}_{{kl}}\end{array}\right]}^{{\boldsymbol{{\prime} }}}$$

$${\rm{and}}\,{{\bf{E}}}_{{\rm{s}},{\rm{kl}}}{\boldsymbol{=}}{\left[\begin{array}{cccccccc}\mu & {\alpha }_{k,1} & {\alpha }_{l,1} & {\alpha }_{k,2} & {\alpha }_{l,2} & {\delta }_{k,3} & {\delta }_{l,3} & {(\alpha \alpha )}_{{kl},3}\end{array}\right]}^{\prime}$$

Due to assumption of the same functional effect, the additive effect for each locus is represented twice in ${{\bf{E}}}_{{\rm{f}},{\rm{kl}}}$.

Matrices ${{\bf{W}}}_{{\rm{f}},{\rm{kl}}}$ and ${{\bf{W}}}_{{\rm{s}},{\rm{kl}}}$ have the dimension of 15 × 8:

$${{\bf{W}}}_{{\rm{f}},{\rm{kl}}}=\left[\begin{array}{cccccccc}{\boldsymbol{1}} & {\bf{J}}\otimes \frac{1}{2}{{\bf{t}}}_{{\rm{k}},1}^{{\rm{a}}} & \frac{1}{2}{{\bf{t}}}_{{\rm{l}},1}^{{\rm{a}}}\otimes {\bf{J}} & {\bf{J}}\otimes \frac{1}{2}{{\bf{t}}}_{{\rm{l}},2}^{{\rm{a}}} & \frac{1}{2}{{\bf{t}}}_{{\rm{l}},2}^{{\rm{a}}}\otimes {\bf{J}} & {\bf{J}}\otimes {{\bf{t}}}_{{\rm{k}},3}^{{\rm{d}}} & {{\bf{t}}}_{{\rm{l}},3}^{{\rm{d}}}\otimes {\bf{J}} & \frac{1}{2}({{\bf{t}}}_{{\rm{l}},1}^{{\rm{a}}}+{{\bf{t}}}_{{\rm{l}},2}^{{\rm{a}}})\otimes \frac{1}{2}({{\bf{t}}}_{{\rm{k}},1}^{{\rm{a}}}+{{\bf{t}}}_{{\rm{k}},2}^{{\rm{a}}})\end{array}\right]$$

$${\rm{and}}\,{{\bf{W}}}_{{\rm{s}},{\rm{kl}}}=\left[\begin{array}{cccccccc}{\boldsymbol{1}} & {\bf{J}}\otimes \frac{1}{2}{{\bf{h}}}_{{\rm{k}},1}^{{\rm{a}}} & \frac{1}{2}{{\bf{h}}}_{{\rm{l}},1}^{{\rm{a}}}\otimes {\bf{J}} & {\bf{J}}\otimes \frac{1}{2}{{\bf{h}}}_{{\rm{k}},2}^{{\rm{a}}} & \frac{1}{2}{{\bf{h}}}_{{\rm{k}},2}^{{\rm{a}}}\otimes {\bf{J}} & {\bf{J}}\otimes {{\bf{h}}}_{{\rm{k}},3}^{{\rm{d}}} & {{\bf{h}}}_{{\rm{l}},3}^{{\rm{d}}}\otimes {\bf{J}} & \frac{1}{2}({{\bf{h}}}_{{\rm{l}},1}^{{\rm{a}}}+{{\bf{h}}}_{{\rm{l}},2}^{{\rm{a}}})\otimes \frac{1}{2}({{\bf{h}}}_{{\rm{k}},1}^{{\rm{a}}}+{{\bf{h}}}_{{\rm{k}},2}^{{\rm{a}}})\end{array}\right]$$

where ${\bf{J}}$ is a 15 × 1 vector with all elements of 1. Elements of 15 × 1 vectors ${{\bf{t}}}_{{\rm{x}},1}^{{\rm{a}}}{\boldsymbol{=}}{{\bf{t}}}_{{\rm{x}},1}{\boldsymbol{\otimes }}{{\bf{J}}}_{2}$ and ${{\bf{t}}}_{{\rm{x}},2}^{{\rm{a}}}{\boldsymbol{=}}{{\bf{J}}}_{1}{\boldsymbol{\otimes }}{{\bf{t}}}_{{\rm{x}},2}$, where $${{\bf{t}}}_{{\rm{x}},1}{\boldsymbol{=}}{\left[\begin{array}{ccc}-1 & 0 & 1\end{array}\right]}^{{\prime} }$$ and $${{\bf{t}}}_{{\rm{x}},2}{\boldsymbol{=}}{\left[\begin{array}{ccccc}-1 & -0.5 & 0 & 0.5 & 1\end{array}\right]}^{{\prime} }$$, and ${{\bf{J}}}_{1}$ and ${{\bf{J}}}_{2}$ are 3 × 1 and 5 × 1 vectors with all elements of 1. The element of 15 × 1 vector ${{\bf{t}}}_{{\rm{x}},3}^{{\rm{d}}}$ is the corresponding covariate ${t}_{x,i3}^{d}$, as in Eq. 9. Elements of 15 × 1 vectors ${{\bf{h}}}_{{\rm{x}},1}^{{\rm{a}}}{\boldsymbol{=}}{{\bf{t}}}_{{\rm{x}},1}^{{\rm{a}}}{\boldsymbol{-}}2{q}_{x,1}$ and ${{\bf{h}}}_{{\rm{x}},2}^{{\rm{a}}}{\boldsymbol{=}}{{\bf{t}}}_{{\rm{x}},2}^{{\rm{a}}}{\boldsymbol{-}}2{q}_{x,2}$. Elements of 15 × 1 vector ${{\bf{h}}}_{{\rm{x}},3}^{{\rm{d}}}$ are constructed based on the covariate ${h}_{x,i3}^{d}$ as in Eq. 10. These covariates are calculated using allele frequencies of inbred lines 1 and 2. Thus, the calculated additive effects ${\alpha }_{x,1}$ depend on the allele frequencies of base populations 1 and 2, and the calculated variance ${\sigma }_{A,1}^{2}$ based on ${\alpha }_{x,1}$ is not the additive variance of population 1, but ${\sigma }_{A,1}^{2}$ is the additive variance of the three-way hybrid population that is explained by the genotype of the parental inbred population 1. Similarly, variance ${\sigma }_{{A}_{2}}^{2}$ is not the additive variance of the inbred population 2.

## Methods

### Simulation of QTL functional effects

In this study, QTL functional effects that could generate populations with desired statistical variance were simulated. These effects are required for the simulation of genetic effects, and thus phenotypes, of all individuals based on their genotypes and environmental setting. We started with the genotypes of the base populations, then simulated functional effects so that the base populations met the desired statistical variance inputs. However, we also needed the descendant populations to meet the desired input variance with unchanged QTL allele frequencies. The descendant population variance can be re-calculated based on simulated functional effect values or re-estimated from simulated genotype and phenotype data with a GBLUP model. The latter is the output for the assessment of simulation method success, as it corresponds to the method used to obtain input parameters using a GBLUP model.

We investigated the following approaches to QTL functional effect simulation:

- functional effect simulation by: (i) the sampling of statistical effects from input distributions, followed by the calculation of functional effects using the NOIA model (SS_NOIA); and (ii) the use of a genetic algorithm to stochastically optimize the QTL functional effects on the condition of statistical inputs (SF_GA);
- calculation of base population variance by: (i) locus and (ii) individual.

Simulation using the SS_NOIA method generates statistical effects by random sampling from defined distributions, similar to the method described by Karaman et al. (2021), and then the calculation of functional effects using the NOIA formula. With the SF_GA method, the algorithm semi-stochastically finds a set of functional effects (priors sampled randomly from defined distributions) that meet the input requirements; this set represents an individual made up of “genes” or functional effect units (Scrucca 2013). The fitness $f$ of individuals in a population is then calculated. With GA evolution, the fittest individuals survive, passing their “genetic information” to their offspring and thereby mimicking natural selection in the population (Scrucca 2013). The GA looks for solutions by creating population diversity using the biological concepts of mutation and crossover (Scrucca 2013). The SS_NOIA method relies on backward transformation of NOIA from statistical to functional effects, whereas the SF_GA method uses forward transformation of NOIA from functional to statistical effects to calculate fitness for the GA. Both methods can be used to simulate functional effects with statistical variance given as inputs, but they could yield different sets of random (SS_NOIA) and pseudorandom (SF_GA) functional effects. The randomness in functional effect generation is the principle of stochastic simulation.

The simulation methods were investigated using three examples with different data types and genome assumptions:

1. iPGs with LE between QTL,
2. iPGs with linkage disequilibrium (LD) between QTL,
3. pPGs from three-way hybrid crop breeding with LD between QTL.

### Example 1: Individual phenotypes with LE

The predictive model used with the iPGs was the same as M.3. The vectors of ${\bf{y}}$, ${\bf{u}}$, ${\bf{v}}$, and $\left({\bf{uu}}\right)$ were individual phenotypes, breeding values, dominance deviations, and epistatic values, respectively. Variance components from the model (${\sigma }_{A}^{2}$, ${\sigma }_{D}^{2}$, and ${\sigma }_{{AA}}^{2}$) were the inputs for the simulation of the functional effects of QTL ${\bf{a}}$, ${\bf{d}}$, and ${\boldsymbol{(}}{\bf{aa}}{\boldsymbol{)}}$. The variance estimates could be considered as simulation outputs, as the input values were obtained in manner similar to real data acquisition. The assumption of LE and independent QTL inheritance ensured that the assumptions of M.3 were met.

We generated a base population (generation 0) of 100 individual genotypes created by random binomial sampling for 2000 QTL (${n}_{{qtl}}=2000$) based on QTL allele frequencies drawn from random uniform distribution between 0.05 and 0.95, for the simulation of additive genetics and dominance. For computational ease, we assumed that epistatic interaction occurred between QTL pairs, with each QTL present in precisely one pair. Thus, epistasis was simulated using 1000 (${n}_{{epi}}=1000$) interaction pairs. We used the base population genotypes to simulate functional genetic effects of QTL with the SS_NOIA and SF_GA methods.

For SS_NOIA:

1. Vectors of starting (or prior) values of statistical additive effects ${{\boldsymbol{\alpha }}}^{* }$ and dominance degree ${{\bf{d}}}_{{\rm{d}}}$ were sampled from normal distributions *N(0*, ${\sigma }_{A}^{2}$*)* and *N(0.19, 0.097)*, respectively (Wellmann and Bennewitz 2011). Based on ${{\bf{d}}}_{{\rm{d}}}$, the vector of the prior statistical dominance values was: ${{\boldsymbol{\delta }}}^{* }={{\bf{d}}}_{{\rm{d}}}\times \left|{{\boldsymbol{\alpha }}}^{* }\right|$. The vector of prior epistatic effect ${{\boldsymbol{(}}{\boldsymbol{\alpha }}{\boldsymbol{\alpha }}{\boldsymbol{)}}}^{* }$ was sampled from N(0, ${\sigma }_{{AA}}^{2}$).
2. Statistical effects ${\boldsymbol{\alpha }}$, ${\boldsymbol{\delta }}$, and ${\boldsymbol{(}}{\boldsymbol{\alpha }}{\boldsymbol{\alpha }}{\boldsymbol{)}}$ were calculated by rescaling prior effects ${{\boldsymbol{\alpha }}}^{* }$, ${{\boldsymbol{\delta }}}^{* }$, and ${{\boldsymbol{(}}{\boldsymbol{\alpha }}{\boldsymbol{\alpha }}{\boldsymbol{)}}}^{* }$ to achieve the desired statistical variance inputs of ${\sigma }_{A}^{2}$, ${\sigma }_{D}^{2}$, and ${\sigma }_{{AA}}^{2}$ for the base population. Given statistical effect of ${{\boldsymbol{\alpha }}}^{* }$, ${{\boldsymbol{\delta }}}^{* }$, and ${{\boldsymbol{(}}{\boldsymbol{\alpha }}{\boldsymbol{\alpha }}{\boldsymbol{)}}}^{* }$, we can compute the prior statistical variances ${\sigma }_{{A}^{* }}^{2}$, ${\sigma }_{{D}^{* }}^{2}$ and ${\sigma }_{{{AA}}^{* }}^{2}$ using the calculation of variance by locus or by individual as all individuals’ genotypes in the base population are known. After that, the rescaling process is done, eg. ${\boldsymbol{\delta }}={{\boldsymbol{\delta }}}^{* }\times \sqrt{\frac{{\sigma }_{D}^{2}}{{\sigma }_{{D}^{* }}^{2}}}$.
3. Using the NOIA model, the functional effects of ${\bf{a}}$, ${\bf{d}}$, and $({\bf{aa}}{\boldsymbol{)}}$ were calculated based on the statistical effects of ${\boldsymbol{\alpha }}$, ${\boldsymbol{\delta }}$, and ${\boldsymbol{(}}{\boldsymbol{\alpha }}{\boldsymbol{\alpha }}{\boldsymbol{)}}$. For example, we considered loci $k$ and $l$:

11 $${{\bf{E}}}_{{\rm{f}},{\rm{kl}}}={({{\bf{W}}}_{{\rm{f}},{\rm{kl}}}^{{\prime} }{{\bf{W}}}_{{\rm{f}},{\rm{kl}}})}^{-1}{{\bf{W}}}_{{\rm{f}},{\rm{kl}}}^{{\prime} }{{\bf{W}}}_{{\rm{s}},{\rm{kl}}}{{\bf{E}}}_{{\rm{s}},{\rm{kl}}}.$$

The notations in Eq. 11 are the same as those in Eq. 7. Allele frequencies and genotypes for ${{\bf{E}}}_{{\rm{f}},{\rm{kl}}}$ calculation were based on the base population genotypes.

The SF_GA method (Scrucca 2013) uses the basic principles of biological evolution to stochastically optimize the solutions through a fitness function. Different potential solutions are explored to find a set of functional effects ${\bf{a}}$, ${{\bf{d}}}_{{\rm{d}}}$, and $({\bf{aa}}{\boldsymbol{)}}$ that meet the requirements of ${\sigma }_{A}^{2}$, ${\sigma }_{D}^{2}$, and ${\sigma }_{{AA}}^{2}$ for the base population. To run the algorithm, we made a fitness function:

- We assumed a potential solution set of functional genetics ${{\bf{a}}}^{* }$, dominance degree ${{\bf{d}}}_{{\rm{d}}}^{* }$, and functional epistasis ${({\bf{aa}}{\boldsymbol{)}}}^{{\boldsymbol{* }}}$. The vector of the dominance values is: ${{\bf{d}}}^{* }={{\bf{d}}}_{{\rm{d}}}^{* }\times \left|{{\bf{a}}}^{* }\right|$.
- We converted functional effects ${{\bf{a}}}^{* }$, ${{\bf{d}}}^{* }$, and ${({\bf{aa}}{\boldsymbol{)}}}^{{\boldsymbol{* }}}$ to statistical effects ${{\boldsymbol{\alpha }}}^{* }$, ${{\boldsymbol{\delta }}}^{* }$, and ${({\boldsymbol{\alpha }}{\boldsymbol{\alpha }}{\boldsymbol{)}}}^{{\boldsymbol{* }}}$ using Eq.7, based on the base population allele frequencies and genotypes.
- From the statistical effects ${{\boldsymbol{\alpha }}}^{* }$, ${{\boldsymbol{\delta }}}^{* }$, and ${({\boldsymbol{\alpha }}{\boldsymbol{\alpha }}{\boldsymbol{)}}}^{{\boldsymbol{* }}}$, we calculated statistical variance values ${\sigma }_{{A}^{* }}^{2}$, ${\sigma }_{{D}^{* }}^{2}$, and ${\sigma }_{{{AA}}^{* }}^{2}$.
- We calculated fitness as: ${f}_{{iPG}}=-\left|{\sigma }_{{A}^{* }}^{2}-{\sigma }_{A}^{2}\right|-\left|{\sigma }_{{D}^{* }}^{2}-{\sigma }_{D}^{2}\right|-\left|{\sigma }_{{{AA}}^{* }}^{2}-{\sigma }_{{AA}}^{2}\right|$.

With the maximation of fitness, (${f}_{{iPG}}=0$), we obtained the solution for the functional effects of ${\bf{a}}$, ${\bf{d}},$ and ${\boldsymbol{(}}{\bf{aa}}{\boldsymbol{)}}$. We calculated the simulating variance (${\sigma }_{A}^{2}$, ${\sigma }_{D}^{2}$, and ${\sigma }_{{AA}}^{2}$) by individual and locus.

In the breeding scheme, the genotypes of individuals in generations 1–4 were generated through independent QTL inheritance following standard Mendelian principles. In each generation, 150 random matings among 150 randomly selected parents occurred. Each mating generated 5 offspring (total, 750 offspring). In total, 3000 individual genotypes were created. These steps were taken to create the genotypes of a population in HWE with no LD between QTL. Based on the genotypes and QTL functional effects, the phenotypes of the 3000 individuals were simulated using M.1. The simulated genotype and phenotype data was used for deriving the calculating variance by individual and by locus. These datasets were also used for variance re-estimation using M.3 and the DMUAI procedure of the DMU package (Madsen and Jensen 2013). The QTL were used as markers in the construction of genomic relationships for variance component estimation. With the combination of simulation methods (SS_NOIA and SF_GA) and calculations of simulating variance (by individual or locus), four different approaches were taken to simulate functional effects in example 1. Each approach was replicated 30 times. For each set of functional effects, breeding scheme (generation 1–4) and variance estimation using M.3 were repeated 8 times.

### Example 2: Individual phenotypes with LD

Simulation procedures in example 2 were the same as those in example 1, except that the genome was simulated with LD between QTL. The base population was generated using a Fisher-Wright inheritance model (Fisher 1930) to create an LD genome consisting of seven 230-cM chromosomes, emulating that of rye (Milczarski et al. 2011). On each chromosome, positions of 50k loci with an allele frequency of 0.5 were sampled from a random uniform distribution. A historical population from generation –1000 to generation –1 (*n* = 1000 each) was simulated with QMSim (Sargolzaei and Schenkel 2009) to establish mutation-drift equilibrium. At generation 0, the base population of 100 individual genotypes, each with 2000 QTL, was created. The QTL were drawn randomly from loci that were segregating with a minor allele frequency ≥ 0.05. As our interest was in true values, we did not simulate markers. The QTL were also used as markers in the construction of genomic relationships for variance component estimation. QTL inheritance patterns from parents to offspring followed standard Mendelian principles, allowing for recombination as in the ADAM software (Pedersen et al. 2009). Other steps, including the sampling of functional QTL effects to meet the base population inputs and the generation of phenotype data for the re-estimation and re-calculation of variance in descendant populations, were performed as in example 1.

### Example 3: Plot phenotypes from three-way hybrid crop breeding

Example 3 was created with pPGs and an LD genome. The predictive model was the same as M.5, with the use of plot phenotypes and inbred-line genotypes from two different populations. The estimated variance values from the model (${\sigma }_{A,1}^{2}$, ${\sigma }_{A,2}^{2}$, ${\sigma }_{D,3}^{2}$, and ${\sigma }_{{AA},3}^{2}$) were the inputs for the simulation of QTL functional effects ${\bf{a}}$, ${\bf{d}}$, and ${\boldsymbol{(}}{\bf{aa}}{\boldsymbol{)}}$.

Genotype simulation began with the formation of two historical inbred populations, the creation of base populations, and the establishment of a three-way crop breeding scheme (Figure of the scheme can be seen in Appendix 3). Inbred population formation and the inheritance pattern and genome structure in example 3 were similar to those in example 2. QMSim (Sargolzaei and Schenkel 2009) was used to form a common historical population from generation –1100 to generation –101, and then diverge it into two populations from generation –100 to generation –1. At generation 0, two base populations of 100 inbred lines each were created after four mini-generations of self-pollination. A base population of 100 two-way hybrids was created by crossing inbred lines of base population 2, with all of these lines contributing equally. A base population of three-way hybrids was created by crossing inbred lines of base population 1 with the two-way hybrids.

For generations 1–4, the simulation of the three-way hybrid breeding scheme was a simplified version of a rye breeding program (Kristensen et al. 2023) with discrete generations and random selection. Population 1 represented restorer lines, and population 2 represented male sterile and non-restorer lines (Kristensen et al. 2023). In both populations, inbred lines were created by self-pollination for four mini-generations, resulting in a mean inbreeding coefficient of 0.938. These lines were then crossed randomly within populations, and five seeds from each cross were kept. The plants that grew from these seeds were self-pollinated four times, with only one seed kept from each self-pollination (single seed descent). The two-way hybrids were produced by random mating of 100 inbred lines at generation *t* with 100 inbred lines at generation *t* – 1 in population 2. The three-way hybrids were created by random mating of 200 inbred lines from population 1 and 100 two-way hybrids, resulting in 1000 matings in each of generations 2–4. Each inbred line contributed to 5 matings, each two-way hybrid contributed to 10 matings, and each mating generated one three-way hybrid (plot phenotype in this paper). In total, the scheme generated 3000 plots from different matings. All mating and individual selection were random to minimize changes in allele frequency in the descendant populations.

The genotypes from the inbred line and hybrid base populations were used to simulate functional effects and calculate true values for statistical effects and variance. In preliminary simulations of QTL functional effects, we used SS_NOIA, SF_GA, and variance calculation approaches. The SS_NOIA method generated different functional effects for the same QTL positions, contrary to our assumption of the same functional effects across populations. Appendix 4 contains an R script example to illustrate SS_NOIA method in case of pPG. No solution was found with the use of the SF_GA method and calculation of simulating variance by locus, even after multiple attempts with prolonged GAs. Thus, we used only the SF_GA method with the calculation of simulating variance by individual to simulate functional effects. This approach was similar to SF_GA method application in example 1, except that the calculation of statistical effects from functional effects was changed for the fitness function.

From a potential SF_GA solution set of functional effects ${{\bf{a}}}^{* }$, ${{\bf{d}}}_{{\rm{d}}}^{* }$, and ${({\bf{aa}}{\boldsymbol{)}}}^{{\boldsymbol{* }}}$, statistical effects ${{\boldsymbol{\alpha }}}_{1}^{{\boldsymbol{* }}}$, ${{\boldsymbol{\alpha }}}_{2}^{* }$, ${{\boldsymbol{\delta }}}_{3}^{* }$, and ${({\boldsymbol{\alpha }}{\boldsymbol{\alpha }}{\boldsymbol{)}}}_{3}^{* }$ were calculated using the NOIA formula, and then statistical variance (${\sigma }_{{A}^{* },1}^{2}$, ${\sigma }_{{A}^{* },2}^{2}$, ${\sigma }_{{D}^{* },3}^{2}$, and ${\sigma }_{{{AA}}^{* },3}^{2}$) was calculated. The fitness function for optimization (${f}_{{pPG}}=0$) was:

$${f}_{{pPG}}=-\left|{\sigma }_{{A}^{* },1}^{2}-{\sigma }_{A,1}^{2}\right|-\left|{\sigma }_{{A}^{* },2}^{2}-{\sigma }_{A,2}^{2}\right|-\left|{\sigma }_{{D}^{* },3}^{2}-{\sigma }_{D,3}^{2}\right|-\left|{\sigma }_{{{AA}}^{* },3}^{2}-{\sigma }_{{AA},3}^{2}\right|.$$

Functional QTL effects ${\bf{a}}$, ${\bf{d}}$, and ${\boldsymbol{(}}{\bf{aa}}{\boldsymbol{)}}$, which were SF_GA solutions, were used to simulate plot phenotypes of three-way hybrids in generations 2–4. These plot phenotypes and the genotypes of inbred lines in populations 1 and 2 were used for variance re-estimation with M.5 and the DMUAI procedure of the DMU package (Madsen and Jensen 2013). These genotype and phenotype datasets were also used for deriving the calculating variance. Functional effect simulation was replicated 30 times. For each set of functional effects, the breeding scheme (generations 1–4) and variance estimation were run eight times to validate the statistical variance output, as in examples 1 and 2.

## Results

Table 2 shows the variance from iPG simulation in example 1. The SS_NOIA method and the SF_GA method with calculation of simulating variance by locus generated equivalent input and output variance for additive genetics and epistasis. However, the output dominant and residual variance did not seem to meet the input variance with these simulation methods; the latter was smaller than the input. The patterns of the simulation results obtained in example 2 were similar to those obtained in example 1 (Table 3). For example, the SS_NOIA method generated equivalent input and output additive variance.

**Table 2:** Example 1—Variance inputs and outputs for data type of individual phenotypes with genome in linkage equilibrium.

	Additive genetics (mean ± SD)	Epistasis (mean ± SD)	Dominance (mean ± SD)	Residual (mean ± SD)
Input values^a^	400	100	100	100
SS_NOIA & simulating variance by locus^b^				
Estimated statistical variance – output^c^	404 ± 23	106 ± 33	114 ± 11	86 ± 26
Calculated statistical variance by individual^d^	400 ± 25	100 ± 3	120 ± 9	100 ± 2
Calculated statistical variance by locus^d^	404 ± 3	100 ± 1	103 ± 1	100 ± 2
Calculated functional variance by individual^e^	494 ± 30	172 ± 8	149 ± 9	100 ± 2
SS_NOIA & simulating variance by individual^b^				
Estimated statistical variance - output^c^	405 ± 60	103 ± 38	118 ± 29	90 ± 27
Calculated statistical variance by individual^d^	405 ± 48	100 ± 16	129 ± 32	100 ± 3
Calculated statistical variance by locus^d^	407 ± 46	99 ± 16	119 ± 27	100 ± 3
Calculated functional variance by individual^e^	503 ± 49	171 ± 26	159 ± 49	100 ± 3
SF_GA & simulating variance by locus^b^				
Estimated statistical variance - output^c^	403 ± 24	104 ± 33	114 ± 11	88 ± 25
Calculated statistical variance by individual^d^	406 ± 27	99 ± 1	121 ± 10	100 ± 3
Calculated statistical variance by locus^d^	404 ± 4	98 ± 3	102 ± 11	100 ± 3
Calculated functional variance by individual^e^	530 ± 31	171 ± 9	151 ± 11	100 ± 3
SF_GA & simulating variance by individual^b^				
Estimated statistical variance - output^c^	493 ± 78	104 ± 37	93 ± 12	88 ± 26
Calculated statistical variance by individual ^d^	482 ± 66	98 ± 16	97 ± 12	99 ± 2
Calculated statistical variance by locus^d^	505 ± 68	82 ± 7	96 ± 16	99 ± 2
Calculated functional variance by individual^e^	396 ± 65	167 ± 27	120 ± 14	99 ± 2

SS_NOIA was a simulation method that sampled statistical effects from input distributions followed by the calculation of functional effects using NOIA model.

SF_GA was a simulation method that used genetic algorithms to stochastically optimize the functional effects on the condition of statistical inputs.

^a^Functional effects were simulated to meet the inputs in the base population.

^b^Calculation method of simulating statistical variance to meet the inputs in the base population was done either by locus or by individual.

^c^The estimating variance was obtained from variance component re-estimation of GBLUP using DMUAI package with data from generations 1–4.

^d^Calculating statistical variance was calculated using data from generations 1–4. Calculation of the variance was by individual or by locus.

^e^Calculating functional variance was calculated using data from generations 1–4. Calculation of the variance was by individual or by locus.

**Table 3:** Example 2—Variance inputs and outputs for data type of individual phenotypes with genome in linkage disequilibrium.

	Additive genetics (mean ± SD)	Epistasis (mean ± SD)	Dominance (mean ± SD)	Residual (mean ± SD)
Input values^a^	400	100	100	100
SS_NOIA & simulating variance by locus^b^				
Estimated statistical variance - output^c^	393 ± 26	102 ± 25	118 ± 10	89 ± 18
Calculated statistical variance by individual^d^	398 ± 31	99 ± 3	128 ± 11	100 ± 3
Calculated statistical variance by locus^d^	403 ± 30	100 ± 1	102 ± 11	100 ± 3
Calculated functional variance by individual^e^	574 ± 40	229 ± 12	170 ± 13	100 ± 3
SS_NOIA & simulating variance by individual^b^				
Estimated statistical variance - output^c^	440 ± 76	106 ± 30	105 ± 15	90 ± 18
Calculated statistical variance by individual^d^	441 ± 64	108 ± 14	115 ± 15	100 ± 2
Calculated statistical variance by locus^d^	444 ± 79	110 ± 13	98 ± 15	100 ± 2
Calculated functional variance by individual^e^	608 ± 72	246 ± 40	153 ± 20	100 ± 2
SF_GA & simulating variance by locus^b^				
Estimated statistical variance - output^c^	403 ± 25	98 ± 26	120 ± 10	91 ± 20
Calculated statistical variance by individual^d^	400 ± 31	100 ± 4	131 ± 11	101 ± 2
Calculated statistical variance by locus^d^	406 ± 3	100 ± 1	102 ± 1	101 ± 2
Calculated functional variance by individual^e^	602 ± 45	228 ± 13	172 ± 12	101 ± 2
SF_GA & simulating variance by individual^b^				
Estimated statistical variance - output^c^	466 ± 60	97 ± 26	94 ± 14	90 ± 19
Calculated statistical variance by individual^d^	445 ± 50	104 ± 21	100 ± 15	100 ± 3
Calculated statistical variance by locus^d^	457 ± 57	104 ± 23	90 ± 10	100 ± 3
Calculated functional variance by individual^e^	304 ± 50	135 ± 20	135 ± 20	100 ± 3

See Table 2 for more explanations of abbreviations and parameters.

In examples 1 and 2, the dominance output exceeded the input variance and the functional additive variance exceeded the statistical variance, except with the use of the SF_GA method and calculation of simulating variance by individual. The estimated variance did not meet the input variance with calculation by locus; the statistical variance for dominance calculated by locus was close to the input value, indicating that the allele and genotype frequencies in the descendant population were the same as those in the base population.

Table 4 shows the variance from pPG simulation using the SF_GA method in example 3. The variance outputs for dominance and epistasis were close to the input values. Variance output ${\sigma }_{A,1}^{2}$ was approximately four times ${\sigma }_{A,2}^{2}$.

**Table 4:** Example 3- Variance inputs and outputs for data type of plot phenotypes from three-way hybrid crop breeding using simulation method SF_GA and calculation of variance by individual.

	Additive genetics variance 1 (${\sigma }_{A,1}^{2}$) (mean ± SD)	Additive genetics variance 2 (${\sigma }_{A,2}^{2}$) (mean ± SD)	Dominance 3 (${\sigma }_{D,3}^{2}$) (mean ± SD)	Epistasis 3 (${\sigma }_{{AA},3}^{2}$) (mean ± SD)	Residual (${\sigma }_{e,3}^{2}$) (mean ± SD)
Input values	200	37.5	50	62.5	100
Estimated statistical variance - output	181 ± 23	47 ± 7	48 ± 7	61 ± 19	101 ± 9
Calculated statistical variances by individual	188 ± 28	47 ± 6	48 ± 2	59 ± 9	100 ± 3
Calculated statistical variances by locus	186 ± 13	50 ± 6	47 ± 5	59 ± 8	100 ± 3
Calculated functional variances by individual	258 ± 25	63 ± 8	86 ± 10	134 ± 22	100 ± 3

${\sigma }_{A,1}^{2}$ and ${\sigma }_{A,2}^{2}$ are additive genetic variances of three-way hybrids explained by the genotypes of inbred line population 1 and 2, respectively; ${\sigma }_{D,3}^{2}$ is the dominance variance of the plots explained by the genotypes of inbred population 1 and 2; ${\sigma }_{{AA},3}^{2}$ is the additive x additive epistasis of the three-way hybrid plots explained by the genotypes of inbred population 1 and 2.

## Discussion

This paper describes methods for the simulation of the functional effects of additive genetics, dominance, and epistasis, with the statistical variance of the effects assumed to be estimated from real data serving as inputs.

Variance was calculated by locus and by individual. The calculation of variance by locus assumed no covariance between loci, as in GBLUP models, but covariance did exist due to LD between QTL. Lara et al. (2022) shows the covariance could be also due to LD between QTL and markers when markers were not QTL. Variance calculation by individual could account for covariance between QTL through the prior summing of effects for all loci in individuals. However, the application of such calculation in a simulation that meets the desired statistical variance in the base population may not yield the desired variance in the descendant population, as specific LD in the base population may break down in the descendant population due to crossover and locus recombination. Examples 1 and 2 in this paper show that simulating variance calculation by locus led to better agreement between the input and output variance.

A limitation of NOIA application in simulation is that the formula does not account for covariance between QTL or between functional effects of additive and dominance. Covariance between QTL is due to LD. The statistical effects of additive and dominance have no covariance due to their orthogonal properties (Álvarez-Castro and Carlborg 2007; Vitezica et al. 2017). However, when the method of Wellmann and Bennewitz (2011) is applied, the two functional effects have positive covariance. To achieve such positive covariance, we simulated dominance effects (${\bf{d}}={{\bf{d}}}_{{\rm{d}}}\times \left|{\bf{a}}\right|$) so that the mean of ${{\bf{d}}}_{{\rm{d}}}$ was positive as in previous studies (Faux et al. 2016; Duenk et al. 2020; Wientjes et al. 2023). This positive covariance leads to greater statistical additive variance than functional variance. However, the greater statistical variance was not achieved with the use of the SS_NOIA method. In example 2, covariance between QTL and between the functional effects of additive and dominance was accounted with the use of the SF_GA method and variance calculation by individual.

In examples 1 and 2, we did not obtain identical input and output values, particularly for dominance and residual variance, most likely due to problems with estimation with GBLUP models. Many of the off-diagonal elements of genomic relationship matrices for dominance and additive × additive epistasis had values close to zero. In other words, these matrices are similar to the residual identity matrix, opening the possibility for confounding and competing effects among the dominance, epistasis, and residual variance components. In the dominance matrix, covariance between individuals is mostly from full-sibs. If the breeding scheme data lacked family structure, the estimation of dominance and epistatic effects would be difficult or lead to very large standard errors. Nonetheless, input and output values overlapped even when the residual variance was biased downward.

In the pPG simulation, the inputs were estimated variance components from M.5. This model assumes a single statistical epistatic effect, in contrast to those of Kristensen et al. (2023) and González-Diéguez et al. (2021). In this study, no additive × dominance, dominance × dominance, or more-than-two-way interaction was simulated for epistasis, such that the functional epistatic effects (${\boldsymbol{\alpha }}{\boldsymbol{\alpha }}$) were equivalent to the statistical effects (${\bf{aa}}$). Thus, M.5 does not include epistasis within and between parental populations, as do the models of Kristensen et al. (2023) and González-Diéguez et al. (2021). Compared with the use of these models, the calculation of the genomic relationship for dominance with M.5 was generalized in one novel formula (Eq. 10). This formula also enables the calculation of dominance relationships for more-than-three-way hybrids. Appendix 5 illustrates dominance relationships of Eq.10 in specific cases of Kristensen et al. (2023) and González-Diéguez et al. (2021). Another notable feature of M.5 is the construction of the epistatic relationship matrix ${{\bf{G}}}_{{{\rm{AA}}}_{3}}$. In the formula ${h}_{j,i3}^{a}=\frac{1}{2}{t}_{j,i1}+\frac{1}{4}\left({t}_{j,i2a}+{t}_{j,i2b}\right)-2{q}_{j,3}$, the genotype codes are not limited to –1, 0, and 1, which enables the calculation of relationships between plots based on parental-line genotype means.

Although the statistical effects ${{\boldsymbol{\alpha }}}_{1}$ and ${{\boldsymbol{\alpha }}}_{2}$ were assumed for the contributions of the two inbred parental populations to the plot phenotype in M.5, we assumed the same functional additive effect ${\bf{a}}$, regardless of the population origin in M.4. In the pPG simulation, M.4 also assumed one functional dominance value for each QTL and one functional additive × additive epistasis element for each QTL interaction pair. In contrast, Kristensen et al. (2023) and González-Diéguez et al. (2021) assumed two functional additive effects for a marker originating from two parental populations, although the models still assumed bi-allelic loci in each of these populations and the same marker chips were used for genotyping. Kristensen et al. (2023) and González-Diéguez et al. (2021) also assumed three functional epistatic effects (two within and one between parental groups) for each marker interaction pair. The assumption of different functional effects for markers that originate from two parental populations may be reasonable because the QTL are unknown, and LD between markers and QTL could differ between the populations. For example, the same marker may link to two different QTL in the parental populations. However, only one functional dominance value per marker was assumed (González-Diéguez et al. 2021; Kristensen et al. 2023), which is inconsistent with the assumptions for additive genetics and epistasis. In addition, we used QTL directly for the simulation of functional effects in this study. In a bi-allelic locus model, a difference in functional effects for the same QTL would be illogical because of the QTL’s origin. Given the assumptions of M.4, the SS_NOIA method was not used for pPG simulation because the calculation of functional effects from statistical effects using the NOIA formula would generate two functional additive effects per QTL.

When the functional additive effect ${\bf{a}}$ is the same across populations, a difference in the corresponding statistical effect between parental populations could be due to population differences in the allele frequency, LD between QTL and markers, and/or the multi-allelic state. However, fixed population differences for these factors were assumed in this study. For example, the base populations were simulated only once, then used for the simulation of different pPG scenarios. In other words, the difference in allele frequency between inbred base populations 1 and 2 was unchanged across the simulated scenarios. We propose the use of the SF_GA method to simulate functional effects meeting the desired inputs of variance ${\sigma }_{A,1}^{2}$ and ${\sigma }_{A,2}^{2}$. It would not be possible to sample the functional effects meeting the variance inputs if only one QTL locus affected the phenotype. The SF_GA method relies on the combination of functional effects assigned to different QTL to achieve the difference between ${\sigma }_{A,1}^{2}$ and ${\sigma }_{A,2}^{2}$. Calculation is based on the base populations if the variance meets the desired inputs. When variance was calculated by individual, but not by locus, the GA found a solution in which the functional effects met the desired inputs. However, the output variance was not met in the descendant populations. The difference between ${\sigma }_{A,1}^{2}$ and ${\sigma }_{A,2}^{2}$ was of a magnitude of about four, equivalent to the factor of relationship coefficients for inbred population 2 and the three-way hybrids *versus* that for inbred population 1 and the hybrid (Appendix 2). In Kristensen et al. (2023), the difference between ${\sigma }_{A,1}^{2}$ and ${\sigma }_{A,2}^{2}$ estimated from real rye data was due not only to the relationship coefficients, but also to other unknown factors, potentially including population differences in private QTL, LD between markers and QTL, and functional additive effects at the same loci.

A limitation of this study is the use of a specific functional model. We did not investigate epistatic interactions of additive × dominance, dominance × dominance, or higher-order interactions. Nevertheless, several studies (Vitezica et al. 2017; Duenk et al. 2020; Wientjes et al. 2023) have consistently shown the capability of NOIA models in translating between functional and statistical effects when the interactions with dominance were included.

## Conclusions

This paper provides theoretical basis, mathematical formulas, and novel methods for simulating functional effects. Different methods in simulating functional effects for additive genetics, dominance and epistasis have been shown to meet the desired inputs of variance components estimated from statistical models. We also simulated a desired difference in statistical additive variances between populations when the functional additive effects were assumed to be the same across population. The simulation model was based on three-way hybrid plot phenotype data from crop breeding. We provided a generalized novel formula for the construction of the dominance relationship matrix for hybrids when the parents’ genotypes are known regardless of the inbreeding level of the parents.

## Supplementary information

Supplementary Materials Appendix 1–5

## Data Availability

For this theoretical and simulation-based study, no real-world datasets were utilized. Instead, simulated datasets were generated using custom code developed by the authors. The code used for generating these simulated datasets is openly accessible on GitHub at the following link: https://bit.ly/3QYZ2of. This repository contains the necessary scripts written in R programming language, along with detailed documentation, to replicate the simulations conducted in this study.
