# Correction: Simulation of functional additive and non-additive genetic effects using statistical estimates from quantitative genetic models

**DOI:** 10.1038/s41437-024-00725-x

**Published:** 2024-10-10

**Authors:** Thinh Tuan Chu, Peter Skov Kristensen, Just Jensen

**Affiliations:** 1 Center for Quantitative Genetics and Genomics, Aarhus University, Aarhus, Denmark; 2 Vietnam National University of Agriculture, Faculty of Animal Science, Trâu Quỳ, Gia Lâm, Hanoi, Vietnam

**Keywords:** Animal breeding, Genetic models, Plant breeding

Correction to: *Heredity* 10.1038/s41437-024-00690-5, published online 31 May 2024

In this article, the two sentences in the Introduction section were incorrectly given as:

However, this approach does not guarantee the same functional effects of QTL across populations, as the authors claimed that it would (Karaman et al. 2021). The assumption that the functional effects of QTL are the same, regardless of origin, appears in the literature (Vitezica et al. 2016; Karaman et al. 2021; Poulsen et al. 2022).

It was not a claim, but it was an assumption by the author in Eiríksson et al. (2021). The corrected sentences are:

However, this approach does not guarantee the same functional effects of QTL across populations. The assumption that the functional effects of QTL are the same, regardless of origin, appears in the literature (Vitezica et al. 2016; Eiríksson et al. 2021; Poulsen et al. 2022).
