# Supplementary material for: Simulation of functional additive and non-additive genetic effects using statistical estimates from quantitative genetic models
Source: Heredity (Edinb). 2024 May 31;133(1):33–42. doi: 10.1038/s41437-024-00690-5 (PMC11222558; doi:10.1038/s41437-024-00690-5)
Supplement: Supplementary file 1 — Supplementary Materials Appendix 1–5 [file 41437_2024_690_MOESM1_ESM.docx]

**Appendix 1: R script example to test NOIA formulas to transform between functional and statistical effects for data type of individual phenotypes in a single population**

Description of script:

- Set up NOIA formula to transform between functional (M.1) and statistical effects (M.2) in the main paper
- NOIA formula of this script refers to Eq. 7; and notations can be found in the main paper
- Matrices for the NOIA formula are: $\mathbf{W}_{f,kl}$ and $\mathbf{W}_{s,kl}$ with a dimension of 9 × 6 (rows x columns); $\mathbf{E}_{f,kl}$ and $\mathbf{E}_{s,kl}$ with a dimension of 6 × 1; (optional) diagonal matrix $\mathbf{D}_{\mathrm{kl}}$ with dimension of 9 × 9
- Inputs required: Either $\mathbf{E}_{f,kl}$ or $\mathbf{E}_{s,kl}$**;** frequencies of genotypes (px) at loci *k* and *l* in the population.

| ############### **Required functions** ###################  #’ Set up functional matrix Wfkl (9x6)  #’ Given input: Vector of tx as possible genotype code at locus x (k or l).  #’ @param tx a vector. In this NOIA formula, tx = c(0,1,2).  #’ @return a matrix. Wfkl (9x6) in this formula  setupWfkl <- function (tx=c(0,1,2)) {  if (length(tx)!=3 \| any(tx<0)\| any(tx>2)) stop ('Wrong input to setupWfkl function.')  ta = matrix(tx-1,nrow=3,ncol=1) # additive covariate  tx[tx>1]=abs(tx[tx>1]-2)  td = matrix(tx,nrow=3,ncol=1) # dominance covariate  Jvec = matrix(1,nrow=3,ncol=1)  Wfkl <- matrix(nrow=3*3,ncol=6)  Wfkl[,1]= 1.0  Wfkl[,2]= kronecker(Jvec,ta); Wfkl[,3]= kronecker(ta,Jvec)  Wfkl[,4]= kronecker(Jvec,td); Wfkl[,5]= kronecker(td,Jvec)  Wfkl[,6]= Wfkl[,2]*Wfkl[,3]  return(Wfkl) }  #’ Function to set up Wx matrix as a component to calculate Wskl matrix. Use Eq. 2 & 3 in the main paper  #’ @param px a vector of genotype frequencies at locus x  #’ @return a matrix (3x3)  setupWxs <- function(px) {  if (round(sum(px),digits=1) !=1) stop ('Wrong input to setupWxs function.')  Wx <- matrix(nrow=3,ncol=3)  Wx[,1]=1.0  Wx[,2]=c((0.0-px[2]-2.0*px[3]),(1.0-px[2]-2.0*px[3]),(2.0-px[2]-2.0*px[3]))  temp1 <- (px[3]+px[1]-((px[1]-px[3])^2))  if (temp1!=0) {  Wx[,3]=c((-2.0*px[2]*px[3])/temp1,( 4.0*px[1]*px[3])/temp1,(-2.0*px[1]*px[2])/temp1)  } else {Wx[,3]=0}  return(Wx) }  #’ Function to set up Wskl matrix (9x6) as in the main paper  #’ @param pk a vector of genotype frequencies at locus k  #’ @param pl a vector of genotype frequencies at locus l  #’ @return a matrix (9x6)  setupWskl <- function(pk,pl) {  if (length(pk)!=3 \| any(pk<0)\| any(pk>1)) stop ('Wrong input to setupWskl function.')  if (length(pl)!=3 \| any(pl<0)\| any(pl>1)) stop ('Wrong input to setupWskl function.')  Wskl <- matrix(nrow=3*3,ncol=6)  Wk=setupWxs(pk); Wl=setupWxs(pl)  temp1=kronecker(Wl[,c(1,2)],Wk[,c(1,2)])  Wskl[,1:3]=temp1[,1:3] # a  temp1=kronecker(Wl[,c(1,3)],Wk[,c(1,3)])  Wskl[,4:5]=temp1[,2:3] # d  Wskl[,6]=kronecker(Wl[,c(2)],Wk[,c(2)]) # aa  return(Wskl) }  #’ Function to set up diagonal matrix Dkl (9x9) as in the main paper  #’ @param pk a vector of genotype frequencies at locus k  #’ @param pl a vector of genotype frequencies at locus l  #’ @return a matrix (9x6)  setupDkl <- function(pk,pl) {  if (length(pk)!=3 \| any(pk<0)\| any(pk>1)) stop ('Wrong input to setupDkl function.')  if (length(pl)!=3 \| any(pl<0)\| any(pl>1)) stop ('Wrong input to setupDkl function.')  pk=matrix(pk,nrow = 3); pl=matrix(pl,nrow = 3)  temp1 <- as.vector(kronecker(pl,pk))  Dkl <- diag(temp1)  return(Dkl) }  ############## **Testing NOIA formulas** ##################  #### Random inputs ####  temp1 = runif(2, min = 0.01, max = 0.49); pk = c(temp1,1-sum(temp1))  temp1 = runif(2, min = 0.01, max = 0.49); pl = c(temp1,1-sum(temp1))  Eskl=matrix(c(1,c(runif(5))),nrow = 6,ncol=1)  #### Default input: Adjustment to ensure invertable matrix. It generally has no effects on results.  invertcrit = 1e-10; diagadd = 1e-8  # Setting up matrices  Wfkl <- setupWfkl(); Wskl <- setupWskl(pk,pl); Dkl <- setupDkl(pk,pl)  #### Calculate Efkl from Eskl  temp1 <- t(Wfkl)%*%Wfkl # Test formula without Dkl  if (det(temp1)<invertcrit) diag(temp1)=diag(temp1)+diagadd  Efkl = EfNoDkl = solve(temp1)%*%t(Wfkl)%*%Wskl%*%Eskl  temp1 <- t(Wfkl)%*%Dkl%*%Wfkl # Test formula with Dkl  if (det(temp1)<invertcrit) diag(temp1)=diag(temp1)+diagadd  Efkl = EfYesDkl = solve(temp1)%*%t(Wfkl)%*%Dkl%*%Wskl%*%Eskl  print(data.frame(Eskl,EfNoDkl,EfYesDkl)) # Show & compare results between formula  #### Calculate Eskl from Efkl  Efkl=matrix(c(1,c(runif(5))),nrow = 6,ncol=1)  temp1 <- t(Wskl)%*%Wskl # Test formula without Dkl  if (det(temp1)<invertcrit) diag(temp1)=diag(temp1)+diagadd  Eskl = EsNoDkl = solve(temp1)%*%t(Wskl)%*%Wfkl%*%Efkl  temp1 <- t(Wskl)%*%Dkl%*%Wskl # Test formula with Dkl  if (det(temp1)<invertcrit) diag(temp1)=diag(temp1)+diagadd  Eskl = EsYesDkl = solve(temp1)%*%t(Wskl)%*%Dkl%*%Wfkl%*%Efkl  print(data.frame(Efkl,EsNoDkl,EsYesDkl)) # Show & compare results between formula |
| --- |

**Appendix 2:**

An example to demonstrate model M.5 for plot phenotypes:

$\mathbf{y}_{\boldsymbol{3}}=\mathbf{1}\mu+\mathbf{Z}_{\boldsymbol{u}_{\boldsymbol{1}}}\mathbf{u}_{\boldsymbol{1}}+\left( \mathbf{Z}_{\boldsymbol{u}_{\boldsymbol{2,a}}}+\mathbf{Z}_{\boldsymbol{u}_{\boldsymbol{2,b}}} \right)\mathbf{u}_{\boldsymbol{2}}+\mathbf{Z}_{\boldsymbol{v}_{\boldsymbol{3}}}\mathbf{v}_{\boldsymbol{3}}+\mathbf{Z}_{\boldsymbol{(uu)}_{\boldsymbol{3}}}\mathbf{(uu)}_{\boldsymbol{3}}+\mathbf{e}_{\boldsymbol{3}}$

| Three-way hybrid | Parent 1 | Grandparent 2a | Grandparent 2b | Plot phenotype |
| --- | --- | --- | --- | --- |
| 31 | 11 | 21 | 25 | 101 |
| 32 | 11 | 22 | 25 | 102 |
| 33 | 11 | 22 | 21 | 103 |
| 34 | 12 | 23 | 25 | 104 |
| 35 | 13 | 24 | 21 | 105 |
| 36 | 13 | 25 | 24 | 106 |

$$\left[ \begin{matrix} 101 \\ 102 \\ 103 \\ 104 \\ 105 \\ 106 \end{matrix} \right]=\left[ \begin{matrix} 1 \\ 1 \\ 1 \\ 1 \\ 1 \\ 1 \end{matrix} \right]\mu+\left[ \begin{matrix} 1 & 0 & 0 \\ 1 & 0 & 0 \\ 1 & 0 & 0 \\ 0 & 1 & 0 \\ 0 & 0 & 1 \\ 0 & 0 & 1 \end{matrix} \right]\left[ \begin{matrix} u_{11} \\ u_{12} \\ u_{13} \end{matrix} \right]+\left[ \begin{matrix} 1 & 0 & 0 & 0 & 1 \\ 0 & 1 & 0 & 0 & 1 \\ 1 & 1 & 0 & 0 & 0 \\ 0 & 0 & 1 & 0 & 1 \\ 1 & 0 & 0 & 1 & 0 \\ 0 & 0 & 0 & 1 & 1 \end{matrix} \right]\left[ \begin{matrix} u_{21} \\ u_{22} \\ u_{23} \\ u_{24} \\ u_{25} \end{matrix} \right]+\left[ \begin{matrix} 1 & \cdots& 0 \\ \vdots& \ddots& \vdots\\ 0 & \cdots& 1 \end{matrix} \right]\left[ \begin{matrix} v_{31} \\ v_{32} \\ v_{33} \\ v_{34} \\ v_{35} \\ v_{36} \end{matrix} \right]+\left[ \begin{matrix} 1 & \cdots& 0 \\ \vdots& \ddots& \vdots\\ 0 & \cdots& 1 \end{matrix} \right]\left[ \begin{matrix} \mathrm{uu}_{31} \\ \mathrm{uu}_{32} \\ \mathrm{uu}_{33} \\ \mathrm{uu}_{34} \\ \mathrm{uu}_{35} \\ \mathrm{uu}_{36} \end{matrix} \right]+\left[ \begin{matrix} e_{101} \\ e_{102} \\ e_{103} \\ e_{104} \\ e_{105} \\ e_{106} \end{matrix} \right]$$

Variance:

$$V(y_{3})=\mathbf{Z}_{\boldsymbol{u}_{\boldsymbol{1}}}\mathbf{G}_{\mathbf{A}_{\boldsymbol{1}}}\boldsymbol{Z}_{\boldsymbol{u}_{\boldsymbol{1}}}^{\boldsymbol{'}}\sigma_{A,1}^{2}+\left( \mathbf{Z}_{\boldsymbol{u}_{\boldsymbol{2,a}}}+\mathbf{Z}_{\boldsymbol{u}_{\boldsymbol{2,b}}} \right)\mathbf{G}_{\mathbf{A}_{\boldsymbol{2}}}\left( \mathbf{Z}_{\boldsymbol{u}_{\boldsymbol{2,a}}}+\mathbf{Z}_{\boldsymbol{u}_{\boldsymbol{2,b}}} \right)^{\boldsymbol{'}}\sigma_{A,2}^{2}+\mathbf{Z}_{\boldsymbol{v}_{\boldsymbol{3}}}\mathbf{G}_{\mathbf{D}_{\boldsymbol{3}}}\boldsymbol{Z}_{\boldsymbol{v}_{\boldsymbol{3}}}^{\boldsymbol{'}}\sigma_{D,3}^{2}+\mathbf{Z}_{\boldsymbol{(uu)}_{\boldsymbol{3}}}\mathbf{G}_{\mathbf{AA}_{\boldsymbol{3}}}\boldsymbol{Z}_{\boldsymbol{(uu)}_{\boldsymbol{3}}}^{\boldsymbol{'}}\sigma_{AA,3}^{2}+\sigma_{e_{3}}^{2}$$

Variances $\sigma_{A,1}^{2}$, $\sigma_{A,2}^{2}$, $\sigma_{D,3}^{2}$ and $\sigma_{AA,3}^{2}$ are the components of plot variances that are explained based on the parental genotypes of inbred lines in populations 1 and 2.

To investigate the sources of variances explained by individuals’ genotypes, plot phenotypes and model, we assume populations 1 and 2 have the same allele frequencies, that inbred lines in populations 1 and 2 are completely inbred and non-related, and that the three-way population has additive, dominance and epistatic variances of $\sigma_{A}^{2}$, $\sigma_{D}^{2}$ and $\sigma_{AA}^{2}$ based on genotypes of three-way hybrid individual plants. The variance components that were explained by genotypes of inbred lines in model M.5 would be:

$$V(y_{3})=\mathbf{Z}_{\boldsymbol{u}_{\boldsymbol{1}}}\mathbf{G}_{\mathbf{A}_{\boldsymbol{1}}}\boldsymbol{Z}_{\boldsymbol{u}_{\boldsymbol{1}}}^{\boldsymbol{'}}\frac{\sigma_{A}^{2}}{2}+\left( \mathbf{Z}_{\boldsymbol{u}_{\boldsymbol{2,a}}}+\mathbf{Z}_{\boldsymbol{u}_{\boldsymbol{2,b}}} \right)\mathbf{G}_{\mathbf{A}_{\boldsymbol{2}}}\left( \mathbf{Z}_{\boldsymbol{u}_{\boldsymbol{2,a}}}+\mathbf{Z}_{\boldsymbol{u}_{\boldsymbol{2,b}}} \right)^{\boldsymbol{'}}\frac{\sigma_{A}^{2}}{8}+\mathbf{Z}_{\boldsymbol{v}_{\boldsymbol{3}}}\mathbf{G}_{\mathbf{D}_{\boldsymbol{3}}}\boldsymbol{Z}_{\boldsymbol{v}_{\boldsymbol{3}}}^{\boldsymbol{'}}\frac{\sigma_{D}^{2}}{2}+\mathbf{Z}_{\boldsymbol{(uu)}_{\boldsymbol{3}}}\mathbf{G}_{\mathbf{AA}_{\boldsymbol{3}}}\boldsymbol{Z}_{\boldsymbol{(uu)}_{\boldsymbol{3}}}^{\boldsymbol{'}}\frac{5\sigma_{AA}^{2}}{8}+\sigma_{e_{3}}^{2}$$

The covariance coefficient of additive genetics between plots from the same parent would be $\frac{1}{2}$ for inbred parents. The coefficient would be $\frac{1}{4}$ for non-inbred parents assuming non-related parents in populations. Therefore, the additive genetic variances of plot that are explained by the genotypes of inbred population 1 and 2 are $\frac{1}{2}*\sigma_{A}^{2}$ and $\frac{1}{2}*\frac{1}{4}*\sigma_{A}^{2}$, respectively. Hence, M.5 could explore a total additive genetic variance of $\frac{\sigma_{A}^{2}}{2}+\left( \frac{\sigma_{A}^{2}}{8}+\frac{\sigma_{A}^{2}}{8} \right)=\frac{3\sigma_{A}^{2}}{4}$. As compared to $\sigma_{A}^{2}$ of three-way population, additive genetic variance of $\frac{\sigma_{A}^{2}}{4}$ is missing. This missing variance might not be explored when individual genotypes and phenotypes of three-way hybrids were unknown.

The covariance coefficient of a plot and itself for dominance effect would be 1.0 if the genotypes of individuals within the plot were known. We could only know exactly the genotype of the gamete that was inherited from inbred lines of population 1 to the three-way offspring. The covariance coefficient of dominance for this gamete is 1.0. However, the genotype of the other gamete that was inherited from inbred lines of population 2 to the three-way offspring was unknown. In addition, the effect of dominance is not additive. Therefore, the contribution of the (other) gamete to the plot variance is zero. Hence, the dominance variance of plot that is explained by the genotypes of the two gametes is $\frac{1}{2}*\sigma_{D}^{2}+\frac{0}{2}*\sigma_{D}^{2}=\frac{\sigma_{D}^{2}}{2}$. As compared to $\sigma_{D}^{2}$ of three-way population, dominance variance of $\frac{\sigma_{D}^{2}}{2}$ is missing. This missing variance might not be explored when individual genotypes and phenotypes of three-way hybrids were unknown.

Similarly, we could only know exactly the genotype of the gamete that was inherited from inbred lines of population 1 to the three-way offspring. The covariance coefficient of epistasis for this gamete is 1.0. The genotype of the other gamete that was inherited from inbred lines of population 2 to the three-way offspring was unknown. However, the epistatic effect is additive, thus the covariance coefficient of epistasis for this other gamete is $\frac{1}{4}$. Therefore, the epistatic variance of plot that is explained by the genotypes of the two gametes is $\frac{1}{2}*\sigma_{AA}^{2}+\frac{1}{2}*\frac{1}{4}*\sigma_{AA}^{2}=\frac{5}{8}\sigma_{AA}^{2}$. As compared to $\sigma_{AA}^{2}$ of three-way population, epistatic variance of $\frac{3\sigma_{AA}^{2}}{8}$ is missing.

With M.5, the missing variances of additive genetics $\frac{\sigma_{A}^{2}}{4}$, dominance $\frac{\sigma_{D}^{2}}{2}$ and epistasis $\frac{3\sigma_{AA}^{2}}{8}$ would go to the permanent environmental effect or the residual if each plot consists of finite number of loci. However, here in this study, these missing variances do not existed in the plot variance. This is because we assumed that each plot consists of infinite number of individuals, that the plot phenotype is the mean of all individuals within the plot, and that parents within inbred populations contribute equally genes to the three-way hybrids (random selection and random mating).

**Appendix 3:**


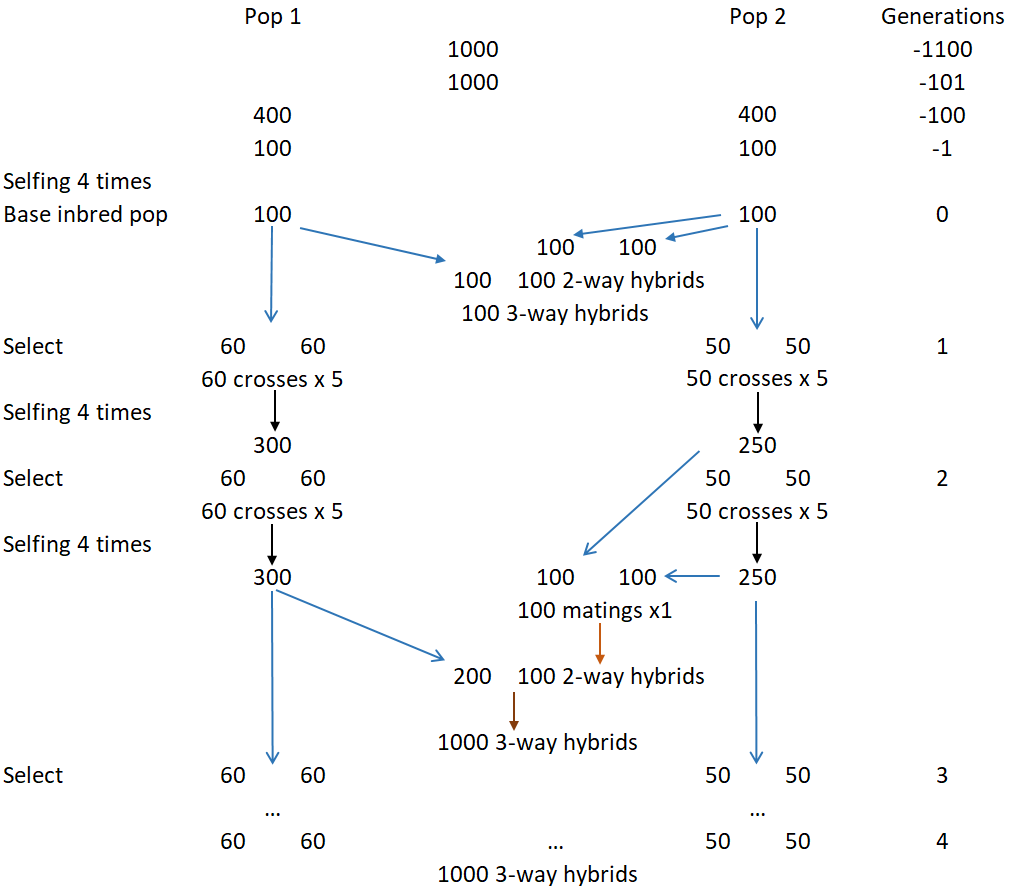


**Figure 1: Historical populations and breeding scheme for three-way hybrids**

**Appendix 4: R script example to test NOIA model for data type of plot phenotypes from crossing of two populations**

Description of script:

- Set up NOIA formula to transform between functional (M.4) and statistical effects (M.5). Notations can be found in the main paper. Matrices in the NOIA formula are: $\mathbf{W}_{f,kl}$ and $\mathbf{W}_{s,kl}$ with a dimension of 15 × 8 (rows x columns); $\mathbf{E}_{f,kl}$ and $\mathbf{E}_{s,kl}$ with a dimension of 8 × 1

Inputs required:

- Either $\mathbf{E}_{f,kl}$ or $\mathbf{E}_{s,kl}$
- Possible genotype codes (tx1, tx2) at locus x (k or l) for parental population 1 and 2. Genotype codes can be mean of genotypes.
- Allele frequencies at loci *k* (p1k, p2k) and *l* (p1l, p2l) in parental population 1 and 2.

| ############### **Required functions** ###################  ##### Set up functional matrice Wfkl in NOIA formula of pPG scenarios.  # Required input:  # Vectors of tx1 & tx2 are possible genotype codes at locus x (k or l) for parental population 1 and 2. Genotype codes can be mean of genotypes. Elements of the vectors can be real or integers within 0 to 2.  setupWfklplot <- function (tx1=c(0,1,2),tx2=c(0,1,2)) {  ngeno1=length(tx1);ngeno2=length(tx2)  if (ngeno1<2 \| any(tx1<0) \| any(tx1>2) \| ngeno2<2 \| any(tx2<0) \| any(tx2>2)) {  stop ('Wrong input to setupWfklplot function.') }  # Calculate functional additive covariates  ta1 = matrix(kronecker(tx1-1,rep(1,ngeno2)),nrow=ngeno1*ngeno2,ncol=1)/2  ta2 = matrix(kronecker(rep(1,ngeno1),tx2-1),nrow=ngeno1*ngeno2,ncol=1)/2  temp1 <- kronecker(tx1,rep(1,ngeno2)); temp2 <- kronecker(rep(1,ngeno1),tx2)  # Use Eq. 9 to calculate functional dominance covariates  td = matrix((1-temp1/2)*(temp2/2) + (temp1/2)*(1-temp2/2),nrow=ngeno1*ngeno2,ncol=1)  Jvec = matrix(1,nrow=ngeno1*ngeno2,ncol=1)  Wfkl <- matrix(nrow=ngeno1*ngeno2*ngeno1*ngeno2,ncol=8)  Wfkl[,1] <- 1.0  Wfkl[,2] <- kronecker(Jvec,ta1); Wfkl[,3] <- kronecker(ta1,Jvec)  Wfkl[,4] <- kronecker(Jvec,ta2); Wfkl[,5] <- kronecker(ta2,Jvec)  Wfkl[,6] <- kronecker(Jvec,td); Wfkl[,7] <- kronecker(td,Jvec)  temp1= (Wfkl[,2:3] + Wfkl[,4:5]); Wfkl[,8] <- temp1[,1]*temp1[,2]  return(Wfkl) }  ##### Set up statistical matrice Wskl in NOIA formula of pPG scenarios.  # Required input:  # Vectors of tx1 & tx2 are possible genotype codes at locus x (k or l) from parental population 1 and 2, respectively. Genotype codes can be mean of genotypes. Elements of the vectors can be real or integers within 0 to 2.  # p1k and p2k are allele frequencies at locus k from parental population 1 and 2. Similarly, for p1l and p2l.  setupWsklPlot <- function(tx1=c(0,1,2),p1k,p2k,tx2=c(0,1,2),p1l,p2l) {  ngeno1=length(tx1); ngeno2=length(tx2)  if (ngeno1<2 \| any(tx1<0) \| any(tx1>2) \| ngeno2<2 \| any(tx2<0) \| any(tx2>2)) {  stop ('Wrong input to setupWsklPlot function.')}  # Use Eq. 2 to calculate statistical additive covariates  ta1k = matrix(kronecker((tx1-2*(1-p1k)),rep(1,ngeno2)),nrow=ngeno1*ngeno2,ncol=1)/2  ta1l = matrix(kronecker((tx1-2*(1-p1l)),rep(1,ngeno2)),nrow=ngeno1*ngeno2,ncol=1)/2  ta2k = matrix(kronecker(rep(1,ngeno1),(tx2-2*(1-p2k))),nrow=ngeno1*ngeno2,ncol=1)/2  ta2l = matrix(kronecker(rep(1,ngeno1),(tx2-2*(1-p2l))),nrow=ngeno1*ngeno2,ncol=1)/2  # Use Eq. 10 to calculate statistical dominance covariates  temp1 <- kronecker(tx1,rep(1,ngeno2)); pa11=1-temp1/2; pa12=temp1/2  temp2 <- kronecker(rep(1,ngeno1),tx2); pa21=1-temp2/2; pa22=temp2/2  temp0=pa11*pa22*2*(1-p1k)*p2k + pa12*pa21*2*p1k*(1-p2k) +  pa11*pa21*(-2)*(1-p1k)*(1-p2k) + pa12*pa22*(-2)*p1k*p2k  tdk = matrix(temp0,nrow=ngeno1*ngeno2,ncol=1)  temp0=pa11*pa22*2*(1-p1l)*p2l + pa12*pa21*2*p1l*(1-p2l) +  pa11*pa21*(-2)*(1-p1l)*(1-p2l) + pa12*pa22*(-2)*p1l*p2l  tdl = matrix(temp0,nrow=ngeno1*ngeno2,ncol=1)  Jvec = matrix(1,nrow=ngeno1*ngeno2,ncol=1)  # Setting up Wskl matrix  Wskl <- matrix(nrow=ngeno1*ngeno2*ngeno1*ngeno2,ncol=8)  Wskl[,1] <- 1.0  Wskl[,2] <- kronecker(Jvec,ta1k); Wskl[,3] <- kronecker(ta1l,Jvec)  Wskl[,4] <- kronecker(Jvec,ta2k); Wskl[,5] <- kronecker(ta2l,Jvec)  Wskl[,6] <- kronecker(Jvec,tdk); Wskl[,7] <- kronecker(tdl,Jvec)  temp1 = (Wskl[,2:3] + Wskl[,4:5]); Wskl[,8] <- temp1[,1]*temp1[,2]  return(Wskl) }  ############## **Testing NOIA formulas** ##################  #### Random inputs ####  Ef=matrix(1,nrow = 8,ncol=1)  Wxk=c(1,rep(runif(1),2),runif(1)); Wxl=c(1,rep(runif(1),2),runif(1))  temp1=kronecker(Wxl[c(1,2)],Wxk[c(1,2)]); Ef[2:3,]=temp1[2:3]  temp1=kronecker(Wxl[c(1,3)],Wxk[c(1,3)]); Ef[4:5,]=temp1[2:3]  temp1=kronecker(Wxl[c(1,4)],Wxk[c(1,4)]); Ef[6:7,]=temp1[2:3]  Ef[8,]= runif(1)  p1k=runif(1, min = 0.01, max = 0.99); p2k=runif(1, min = 0.01, max = 0.99)  p1l=runif(1, min = 0.01, max = 0.99); p2l=runif(1, min = 0.01, max = 0.99)  #### Default input ####  # Adjustment to ensure invertable matrix. It generally has no effects on results.  invertcrit = 1e-10 ; diagadd = 1e-8  #### Setting up matrices  Wfkl=setupWfklplot(tx1=c(0,1,2),tx2=c(0,0.5,1,1.5,2))  Wskl=setupWsklPlot(tx1=c(0,1,2),p1k,p2k,tx2=c(0,0.5,1,1.5,2),p1l,p2l)  #### Calculate Es from Ef  temp1 <- t(Wskl)%*%Wskl  if (det(temp1)<invertcrit) diag(temp1)=diag(temp1)+diagadd  Es=solve(temp1)%*%t(Wskl)%*%Wfkl%*%Ef  print(data.frame(Ef,Es))  #### Calculate Ef from Es  Es=matrix(c(1,c(runif(7))),nrow = 8,ncol=1)  temp1 <- t(Wfkl)%*%Wfkl  if (det(temp1)<invertcrit) diag(temp1)=diag(temp1)+diagadd  Ef=solve(temp1)%*%t(Wfkl)%*%Wskl%*%Es  print(data.frame(Ef,Es)) |
| --- |

**Appendix 5: Elements of dominance relationship matrix for model M.5 analyzing three-way hybrid plot phenotype data**

We illustrate Eq.10 that is equivalent to elements of dominance relationship matrix in Kristensen et al., (2023) and González-Diéguez et al., (2021). Eq.10 for a locus corresponding to these two studies is as follows:

$$h_{3}^{d}=\frac{1}{2}\left( 1-t_{1} \right)\left( t_{2}+1 \right)q_{1}p_{2}+\frac{1}{2}\left( t_{1}+1 \right)\left( 1-t_{2} \right)p_{1}q_{2}-\frac{1}{2}\left( 1-t_{1} \right)\left( 1-t_{2} \right)q_{1}q_{2}-\frac{1}{2}\left( t_{1}+1 \right)\left( t_{2}+1 \right)p_{1}p_{2}$$

Simplification of Eq.10 to specific case as in Kristensen et al., (2023) and González-Diéguez et al., (2021) assuming complete inbred lines:

| Parental genotype | B_1_B_1_ ($t_{1}=-1$) | B_1_b_1_ ($t_{1}=0$) | b_1_b_1_ ($t_{1}=1$) |
| --- | --- | --- | --- |
| B_2_B_2_ x B_2_B_2_ ($t_{2}=-1$) | $-2q_{1}q_{2}$ | $q_{2}(2p_{1}-1)$ | $2p_{1}q_{2}$ |
| B_2_B_2_ x B_2_b_2_ ($t_{2}=-\frac{1}{2}$) | $\frac{1}{2}q_{1}\left( 4p_{2}-3 \right)$ | $-\frac{1}{4}\left( 2p_{1}-1 \right)\left( 4p_{2}-3 \right)$ | $\frac{1}{2}p_{1}({4q}_{2}-1)$ |
| B_2_B_2_ x b_2_b_2_ ($t_{2}=0$) | $q_{1}(2p_{2}-1)$ | $\frac{1}{2}\left( 2p_{1}-1 \right)(2q_{2}-1)$ | $p_{1}(2q_{2}-1)$ |
| B_2_b_2_ x B_2_b_2_ ($t_{2}=0$) | $q_{1}(2p_{2}-1)$ | $\frac{1}{2}\left( 2p_{1}-1 \right)(2q_{2}-1)$ | $p_{1}(2q_{2}-1)$ |
| B_2_b_2_ x b_2_b_2_ ($t_{2}=\frac{1}{2}$) | $\frac{1}{2}q_{1}(4p_{2}-1)$ | $\frac{1}{4}\left( 2q_{1}-1 \right)\left( 4p_{2}-1 \right)$ | $\frac{1}{2}p_{1}(1-4p_{2})$ |
| b_2_b_2_ x b_2_b_2_ ($t_{2}=1$) | $2q_{1}p_{2}$ | $p_{2}\left( 2q_{1}-1 \right)$ | $-2p_{1}p_{2}$ |
